# Safety and effectiveness of ataluren in patients with nonsense mutation DMD in the STRIDE Registry compared with the CINRG Duchenne Natural History Study (2015–2022): 2022 interim analysis

**DOI:** 10.1007/s00415-023-11687-1

**Published:** 2023-04-28

**Authors:** Eugenio Mercuri, Andrés Nascimento Osorio, Francesco Muntoni, Filippo Buccella, Isabelle Desguerre, Janbernd Kirschner, Már Tulinius, Maria Bernadete Dutra de Resende, Lauren P. Morgenroth, Heather Gordish-Dressman, Shelley Johnson, Allan Kristensen, Christian Werner, Panayiota Trifillis, Erik K. Henricson, Craig M. McDonald, the STRIDE and CINRG DNHS investigators

**Affiliations:** 1 Department of Pediatric Neurology, Catholic University, Rome, Italy; 2 Centro Clinico Nemo, Fondazione Policlinico Agostino Gemelli IRCCS, Rome, Italy; 3 Neuromuscular Unit, Department of Neurology and Research in Neuromuscular Diseases, Institut de Recerca Sant Joan de Déu, Center for Biomedical Research Network on Rare Diseases (CIBERER), ISCIII, Barcelona, Spain; 4 UCL Great Ormond Street Institute of Child Health, London, UK; 5 National Institute for Health Research, Great Ormond Street Institute of Child Health Biomedical Research Centre, University College London, London, UK; 6 Parent Project APS, Rome, Italy; 7 Hôpital Necker-Enfants Malades, Paris, France; 8 Department of Neuropediatrics and Muscle Disorders, Medical Center-University of Freiburg, Faculty of Medicine, Freiburg, Germany; 9 Department of Pediatrics, Gothenburg University, Queen Silvia Children’s Hospital, Gothenburg, Sweden; 10 Department of Neurology, Faculty of Medicine, University of São Paulo, São Paulo, SP Brazil; 11 Therapeutic Research in Neuromuscular Disorders Solutions (TRiNDS), Pittsburgh, PA USA; 12 Center for Genetic Medicine, Children’s National Health System and the George Washington, Washington, DC USA; 13 PTC Therapeutics Inc., South Plainfield, NJ USA; 14 PTC Therapeutics Germany GmbH, Frankfurt, Germany; 15 University of California Davis School of Medicine, Davis, CA USA

**Keywords:** Ataluren, Effectiveness, Nonsense mutation Duchenne muscular dystrophy, Safety, STRIDE Registry

## Abstract

**Objective:**

Strategic Targeting of Registries and International Database of Excellence (STRIDE) is an ongoing, international, multicenter registry of real-world ataluren use in individuals with nonsense mutation Duchenne muscular dystrophy (nmDMD) in clinical practice. This updated interim report (data cut-off: January 31, 2022), describes STRIDE patient characteristics and ataluren safety data, as well as the effectiveness of ataluren plus standard of care (SoC) in STRIDE versus SoC alone in the Cooperative International Neuromuscular Research Group (CINRG) Duchenne Natural History Study (DNHS).

**Methods:**

Patients are followed up from enrollment for at least 5 years or until study withdrawal. Propensity score matching was performed to identify STRIDE and CINRG DNHS patients who were comparable in established predictors of disease progression.

**Results:**

As of January 31, 2022, 307 patients were enrolled from 14 countries. Mean (standard deviation [SD]) ages at first symptoms and at genetic diagnosis were 2.9 (1.7) years and 4.5 (3.7) years, respectively. Mean (SD) duration of ataluren exposure was 1671 (56.8) days. Ataluren had a favorable safety profile; most treatment-emergent adverse events were mild or moderate and unrelated to ataluren. Kaplan–Meier analyses demonstrated that ataluren plus SoC significantly delayed age at loss of ambulation by 4 years (*p* < 0.0001) and age at decline to %-predicted forced vital capacity of < 60% and < 50% by 1.8 years (*p* = 0.0021) and 2.3 years (*p* = 0.0207), respectively, compared with SoC alone.

**Conclusion:**

Long-term, real-world treatment with ataluren plus SoC delays several disease progression milestones in individuals with nmDMD. NCT02369731; registration date: February 24, 2015.

**Supplementary Information:**

The online version contains supplementary material available at 10.1007/s00415-023-11687-1.

## Introduction

Duchenne muscular dystrophy (DMD) is a rare, progressive, X-linked neuromuscular disorder characterized by muscle weakness, damage and degeneration [1]. DMD is caused by mutations within the dystrophin (*DMD*) gene that prevent the production of functional dystrophin protein in muscle [1]. Individuals with DMD experience declining muscle function, loss of ambulation, respiratory complications and cardiomyopathy, leading to early death by the second to fourth decade of life depending on clinical management and phenotypic severity [1, 2]. DMD occurs in approximately one in every 3500–5000 live male births [3, 4]. In 10–15% of cases, DMD is caused by a nonsense mutation in the *DMD* gene that results in the production of truncated, non-functional dystrophin [5].

Ataluren is a first-in-class oral treatment for individuals with nonsense mutation DMD (nmDMD) and is designed to promote the synthesis of full-length dystrophin through ribosomal readthrough of an in-frame premature stop codon caused by a nonsense mutation in the dystrophin mRNA [6–8]. Ataluren is indicated for the treatment of ambulatory individuals with nmDMD aged 2 years or older in the European Member States and Belarus, Brazil, Great Britain, Iceland, Israel, Kazakhstan, Liechtenstein, Northern Ireland, Norway, Republic of Korea and Russia, and aged 5 years and older in Chile, the Kingdom of Saudi Arabia and Ukraine (under special state registration) [6]. In Brazil, the indication is restricted to pediatric male patients aged 2 years or older. The presence of a nonsense mutation in the *DMD* gene should be determined by genetic testing [6].

Strategic Targeting of Registries and International Database of Excellence (STRIDE) (ClinicalTrials.gov identifier: NCT02369731) is an ongoing, international, observational, post-approval safety study of ataluren in individuals with nmDMD [9, 10]. The STRIDE Registry was initiated to fulfill a post-marketing commitment to the Pharmacovigilance Risk Assessment Committee of the European Medicines Agency, and to evaluate the long-term safety and effectiveness of ataluren in individuals with nmDMD in real-world routine clinical practice [9, 10]. The Cooperative International Neuromuscular Research Group (CINRG) Duchenne Natural History Study (DNHS) (ClinicalTrials.gov identifier: NCT00468832) was a prospective, longitudinal, observational study of over 400 participants with DMD which enrolled patients between 2006 and 2016 [11, 12].

Interim demographics, safety, and effectiveness results from a previous data cut-off on July 9, 2018 have been published previously [9, 10].

This report evaluates the updated long-term safety and effectiveness results from the STRIDE Registry as of the most recent data cut-off (January 31, 2022), 3.5 years after the previous registry data cut-off date [9, 10]. The demographics and ataluren safety results of the latest STRIDE population are described and the effectiveness results from propensity-score matched patients from STRIDE and the CINRG DNHS are compared.

## Patients and methods

### Study design and methodology

Study design and data collection methodology for the STRIDE Registry and CINRG DNHS have been described in full previously [9, 11, 12]. Briefly, the STRIDE Registry is an ongoing post-approval safety study of ataluren use in real-world clinical practice that assesses individuals with nmDMD in countries where ataluren is commercially available or in those who have received this drug as part of an early access program. The STRIDE Registry began enrolling patients in March 2015 and patients are followed up for at least 5 years from the date of their enrollment or until study withdrawal or death [9, 10]. Patients have been enrolled from 14 countries with 66 active study sites.

The STRIDE Registry originally aimed to enroll approximately 200 patients; enrollment was expected to end upon study entry of the 200th patient or upon completion of a 2-year enrollment period [9]. This target sample size was based upon the number of individuals with nmDMD in the European Union and was consequently chosen for both practical and statistical reasons [9]. The 200th patient was enrolled in February 2018. Because the DMD population available for enrollment in the STRIDE Registry was larger than originally expected, the target sample size was later increased to 270 patients and the follow-up period extended to 5 years from the date of enrollment [9]. In August 2018, the protocol was also amended to include enrollment of at least 20 patients between ≥ 2 and < 5 years of age with the same follow-up period of at least 5 years from the date of enrollment. Furthermore, in March 2020, the sample size limit was further increased to enable enrollment of up to 360 patients; enrollment of patients was extended beyond Europe at this point and included patients from Brazil. Patients or their parents/guardians provided written or informed consent for enrollment in the registry; the study is conducted in accordance with the ethical principles outlined in the Declaration of Helsinki.

The CINRG DNHS enrolled participants with DMD aged 2–28 years from 20 sites in nine countries between 2006 and 2016 [10–12]. Data from participants in the CINRG DNHS who received SoC treatment for DMD (i.e., corticosteroid treatment and supportive therapies) were used as a control for assessing the effectiveness of ataluren in individuals with nmDMD receiving ataluren plus SoC in the STRIDE Registry. Supportive therapies include dietary services, respiratory care, case management, mental health management, decision-making counseling as DMD progresses, legal planning and other supportive services [13].

### Patient eligibility

#### STRIDE Registry

Information regarding patient eligibility for the STRIDE Registry was described previously [9]. In brief, patients are eligible to enroll in the STRIDE Registry if they are, or will be, receiving ataluren (40 mg*/*kg*/*day; 10, 10 and 20 mg*/*kg doses for morning, midday and evening, respectively) through a commercial supply or within an early-access program, have a confirmed genetic diagnosis of nmDMD and are willing to provide written informed consent (either by the patient or through a parent/legal guardian) for data collection. Patients are not eligible if they are receiving ataluren or placebo in an ongoing, blinded, randomized clinical trial or ataluren in any other ongoing clinical trial or early-access program that prevents participation in the STRIDE Registry; however, such patients can become eligible once they have completed the required follow-up and fulfilled the conditions of any trials or programs they are enrolled in. The data cut-off for inclusion in these present analyses was on January 31, 2022; 307 patients were enrolled as of this data cut-off.

#### CINRG DNHS

Eligibility for inclusion in the CINRG DNHS has been described elsewhere [10–12]. Patients aged 2–28 years were required to have a confirmed DMD diagnosis, and those aged 5–28 years must also have had documented clinical symptoms consistent with DMD [11, 12]. Patients did not require an nmDMD diagnosis to be enrolled in the CINRG DNHS, as is the case in the STRIDE Registry; patients with any mutation causing DMD were eligible if they satisfied all other eligibility criteria. Patients were excluded if they were naive to glucocorticoid treatment [11, 12]. In the CINRG DNHS, 440 patients were enrolled. Patients who had received investigational drugs for DMD in previous clinical trials were excluded from the present analyses. Non-ambulatory patients from the CINRG DNHS were defined as those who required full-time use of a wheelchair; the same definition was used for non-ambulatory patients in the STRIDE Registry.

### Statistical analyses

#### STRIDE populations

**Fig. 1:**
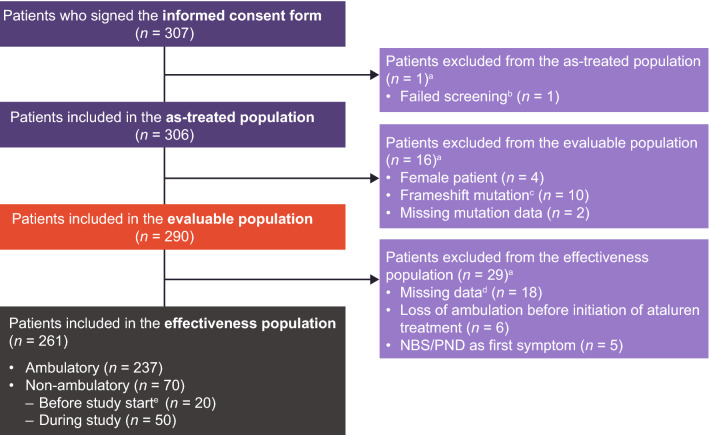
Patient disposition in the STRIDE Registry analysis populations. *DMD* Duchenne muscular dystrophy, *NBS* newborn screening, *PND* prenatal diagnosis, *STRIDE* Strategic Targeting of Registries and International Database of Excellence. ^a^Patients may have been grouped in more than one category. ^b^Screening failure owing to a frameshift mutation. ^c^Ataluren is not indicated in these patients; ataluren is indicated for the treatment of ambulatory patients with DMD resulting from a nonsense mutation in the dystrophin gene. Patients who do not have a nonsense mutation should not receive ataluren [6]. ^d^Data were missing for age at loss of ambulation or age at first symptoms. ^e^Non-ambulatory patients were defined as such if using a wheelchair full-time or bedridden; patients who were non-ambulatory prior to study start were all ambulatory at ataluren initiation in previous clinical trials. Patient disposition for patients aged ≥ 2 to < 5 years of age and ≥ 5 year of age are included in Supplementary Figs. 5 and 6, respectively

The as-treated population is defined as all patients in the STRIDE Registry who provided informed consent and received at least one dose of ataluren; consequently, this population was used for the analysis of safety data; see Fig. 1 for a schematic detailing STRIDE Registry analysis populations. The evaluable population is a subset of the as-treated population who were male and did not fail screening (e.g., owing to missing or incorrect mutation data); this population was used for the analysis of demographics data. Effectiveness analyses were performed using the effectiveness population, which is the subset of the evaluable population excluding those with: newborn screening or a prenatal diagnosis as the first ‘symptom’ recorded; missing data for age at first symptoms; no recorded corticosteroid initiation date, loss of ambulation before the initiation of ataluren, and missing data for age at loss of ambulation. The effectiveness population required no specific duration of exposure to ataluren making analyses of a treatment effect more conservative.

#### Safety and clinical laboratory data in the STRIDE Registry

Adverse events (AEs) were coded using the Medical Dictionary for Regulatory Activities (version 20.1). A treatment-emergent AE (TEAE) is defined as an AE that started on or after the date of first ataluren treatment or worsened after initiation of ataluren treatment and does not necessarily have a causal relationship with ataluren. Clinical laboratory electrocardiogram (ECG) and heart rhythm evaluations were recorded, as well as hypertension status and resting pulse, and the clinical significance of these results was determined by the patient’s clinician. Whether patients’ laboratory evaluations shifted (e.g., from clinically normal to clinically abnormal) was determined using their first available assessment value and establishing their worst case value (i.e., extreme assessment) during ataluren treatment. The extreme assessment was defined as the worst case from the first available assessment during ataluren treatment.

#### STRIDE Registry and CINRG DNHS data comparisons

##### Propensity score matching

Propensity score matching was performed to identify patients in the CINRG DNHS who were comparable to patients in the STRIDE Registry with respect to established predictors of disease progression: age at first symptom onset, age at first corticosteroid use, duration of deflazacort use, and duration of other corticosteroid use [10].

The methodology of propensity score matching has been described in full previously [10]. Propensity score matching is a statistical technique used to reduce bias when assessing treatment effectiveness in observational studies that lack an internal control group [14]. A subgroup of patients from an external comparator group who have similar predefined characteristics as those in the study population (such as those from a natural history study) are matched in an attempt to mimic the randomization process in randomized clinical trials. By matching patients according to these predefined characteristics, confounding factors can be minimized so that outcomes can be more confidently attributed to the treatment rather than any other differences between the groups [14]. Please find a short animation describing the propensity score matching methodology at the following URL: https://www.biomedicine.video/animated-videos/propensity-score-matching-methodology-why-and-how-it-is-used [15].

##### Age-at-event endpoints

For propensity-score matched STRIDE and CINRG DNHS patients, Kaplan–Meier estimates and Cox regression were used to estimate the distribution of the following endpoints: age at loss of ambulation and age when percent predicted forced vital capacity (FVC) was less than 60%, 50%, and 30% and age when FVC was less than 1 L. Age at loss of ambulation has been shown to be predictive of age at onset of loss of upper limb milestones and pulmonary complications [11]. Patients with a predicted FVC of less than 60% are considered to have lung volume restriction that is considered clinically relevant and should receive lung volume recruitment treatment [16]. Those with a predicted FVC of less than 50% are considered in the late non-ambulatory stage of disease progression [10]; manual or mechanically assisted coughing and nocturnal assisted ventilation is strongly recommended [16]. Patients with a predicted FVC of less than 30% are considered to have severe respiratory insufficiency and ventilation is necessary [16, 17]. An FVC below a threshold of 1 L is strongly predictive of mortality within approximately 3 years and is associated with a fourfold increase in the risk of death [11, 18].

Patients who reached the event milestone before their first assessment in the study were not included in these analyses; if they did not reach the event milestone, they were censored at the age of their last assessment. The age-at-event milestone for patients who reached the event was defined as the age at the first time they reached the event. Distribution of the variables was compared between the STRIDE Registry and CINRG DNHS populations using a log-rank test stratified by the duration of deflazacort use and the duration of other corticosteroid use (< 1 month, ≥ 1 to < 12 months, ≥ 12 months). The corticosteroid durations were calculated up to the time at loss of ambulation or the latest time that the patient was still known to be ambulatory minus any duration of corticosteroid cessation by the patient; the overlap of different corticosteroid use was not double counted. The hazard ratio (STRIDE vs CINRG DNHS) and the corresponding 95% confidence interval (CI) were calculated using a Cox proportional hazard model stratified by durations of corticosteroid use, with study (STRIDE or CINRG DNHS), age at first symptoms and age at initiation of corticosteroid use as covariates.

##### Pulmonary function slope analyses in non-ambulatory patients

For propensity-score matched non-ambulatory STRIDE and CINRG DNHS patients, the annual rate of predicted FVC decline, as well as age at loss of ambulation, was assessed in those with a baseline-predicted FVC of more than or equal to 30% and less than or equal to 80%. Annual rate of decline is defined as the difference between the last and the first assessments divided by number of days in-between and multiplied by 365.25. Patients without a second assessment, or for whom the duration between the first and last assessments were less than 48 weeks apart, were excluded from the analysis. Annual rate of pulmonary decline was conducted using pairwise comparisons of FVC (%) and age at the assessment observations. If patients in both the STRIDE Registry and the CINRG DNHS had multiple observations more than 48 weeks after the first assessment, all observations were plotted and analyzed.

The non-ambulatory STRIDE population is defined as the subset of the effectiveness population who were full-time wheelchair users or bedridden either on or before their first recorded commercial or early-access program ataluren use (i.e., prior to their inclusion in the STRIDE Registry) or those who became full-time wheelchair users or bedridden at any time during the STRIDE Registry. Patients who became non-ambulatory during the STRIDE study are included in both the ambulatory and non-ambulatory populations. To be eligible for propensity score matching in these analyses, patients from both the STRIDE Registry and CINRG DNHS must have been non-ambulatory at their last assessment (but could be ambulatory or non-ambulatory at first assessment); the duration between their first and last assessments had to be greater than or equal to 48 weeks; and they had to have a predicted FVC of 30–80% at first assessment. STRIDE patients must have received ataluren for more than or equal to 12 months on their first assessment.

Propensity score matching was performed to identify non-ambulatory CINRG DNHS patients who were comparable to STRIDE patients in the effectiveness population using the following five covariates: age at first symptoms; age at first corticosteroid use; duration of deflazacort use up to the last assessment (i.e., with a predicted FVC more than or equal to 30%); duration of other steroid use up to the last assessment (i.e., with a predicted FVC more than or equal to 30%); and time from loss of ambulation to the last assessment.

##### BMI analyses of STRIDE Registry and CINRG DNHS patients

For propensity-score matched STRIDE and CINRG DNHS patients, body mass index (BMI), BMI *z*-score and BMI percentile at the first and most extreme assessment were analyzed. Propensity score matching was performed to identify patients in the CINRG DNHS who were comparable to patients in the STRIDE Registry at baseline in the following parameters: age; time to stand from supine; duration of deflazacort use; and duration of other corticosteroid use.

A subset of this propensity-score matched sample was used for these analyses, which comprised patients with at least two BMI percentile assessments and those with their first and last assessments at least 40 weeks apart. Shift tables of BMI values were constructed to summarize change in frequency of patients across specified categories of body weight. The shift change in body weight was assessed during the ambulatory stage; for non-ambulatory patients, the last assessment was no later than loss of ambulation.

## Results

### STRIDE Registry patient disposition

The first patient was enrolled in the STRIDE Registry on March 31, 2015. As of January 31, 2022, 307 patients (Fig. 1) from 14 countries with 66 active study sites have been enrolled. The number of active countries, number of study sites and distribution of patients per country is shown in Supplementary Table 1 and Supplementary Fig. 1.

Of the 307 patients who provided informed consent, one patient failed screening owing to having a frameshift mutation; the remaining 306 patients received at least one dose of ataluren and were included in the as-treated population. Of the 306 patients in the as-treated population, four female patients, 10 patients with frameshift mutations (i.e., not nonsense mutations) and two patients with missing mutation data were excluded from the evaluable population (*n* = 290).

Of the 290 patients in the evaluable population, the following 29 patients were excluded from the effectiveness population (*n* = 261): 18 were excluded owing to missing data for age at loss of ambulation or age at first symptoms**,** five were excluded because their first symptom was recorded as ‘newborn screening’ or ‘prenatal diagnosis’ and six were excluded because they started receiving ataluren after loss of ambulation.

The effectiveness population included 237 ambulatory patients. Of the 70 patients in the effectiveness population defined as non-ambulatory, 20 were non-ambulatory prior to study start but ambulatory at ataluren initiation, and 50 lost ambulation during the study. Patients who lost ambulation during the study were double counted.

There were 284 patients in the as-treated population and 268 patients in the evaluable population who were aged 5 years or older. The analyses reported here focus on the safety and demographics data for these patients, given that they had received ataluren for a longer period of time and were part of a larger population than patients aged ≥ 2 to < 5 years, enabling statistically meaningful analyses. Thirty-one patients discontinued the study and thirty-nine stopped ataluren treatment (patients aged 5 years or older in the evaluable population). The reasons for study/ataluren discontinuation are shown in Supplementary Fig. 2.

Of the 440 enrolled CINRG DNHS participants, data from 398 patients were used for effectiveness comparisons with those from patients in the STRIDE Registry. Of the 40 excluded patients in the CINRG DNHS, 22 patients were excluded because they had participated in clinical trials of ataluren or had received eteplirsen, drisapersen or tadalafil; additionally, a further 20 patients were excluded because they had missing data for age at loss of ambulation and age at first symptoms.

### Patient demographics and baseline characteristics

#### Demographics of patients in the STRIDE evaluable population

The demographics and baseline characteristics of patients aged 5 years or older in the evaluable population of the STRIDE Registry are shown in Table 1 and Supplementary Table 2. Equivalent data for patients aged ≥ 2 to < 5 years are shown in Supplementary Table 3. The mean (SD) weight, height and BMI at baseline were 31.7 (14.1) kg, 123.9 (15.8) cm and 19.4 (4.5) kg*/*m^2^, respectively. The mean (SD) age at first symptoms, at time of muscle biopsy, and at genetic confirmation of nmDMD diagnosis was 2.9 (1.7), 4.6 (2.4) and 5.4 (3.1) years, respectively. Of the 268 patients in this population, 155 (57.8%) had a muscle biopsy. The mean age (SD) of patients at informed consent was 10.7 (4.0) years. Patients first captured in the STRIDE Registry had a mean (SD) age of 10.8 (4.0) years. The mean (SD) age of patients at the data cut-off date of January 31, 2022, was 14.8 (3.9) years.

**Table 1:** Demographics and baseline characteristics of patients aged 5 years or older in the STRIDE Registry evaluable population

	All patients
	*N* = 268
Baseline^a^ weight, kg	
*n*	226
Mean (SD)	31.7 (14.1)
95% CI	29.8, 33.6
Median	27.5
Min, max	13.5, 87.0
Baseline^a^ height, cm	
*n*	191
Mean (SD)	123.9 (15.8)
95% CI	121.6, 126.1
Median	121.1
Min, max	94.3, 178.0
Baseline^a^ BMI, kg/m^2^	
*n*	191
Mean (SD)	19.4 (4.5)
95% CI	18.7, 20.0
Median	18.2
Min, max	13.0, 40.1
Age at first symptoms, years	
*n*	253
Mean (SD)	2.9 (1.7)
95% CI	2.7, 3.1
Median	3.0
Min, max	0.1, 8.0
Age at muscle biopsy, years	
*n*	155
Mean (SD)	4.6 (2.4)
95% CI	4.2, 5.0
Median	4.3
Min, max	0.4, 13.0
Age at genetic confirmation of nmDMD diagnosis, years	
*n*	259
Mean (SD)	5.4 (3.1)
95% CI	5.0, 5.8
Median	5.0
Min, max	0.0^b^, 23.0
Previously enrolled in ataluren clinical trial, *n* (%)	
No	197 (73.5)
Yes	71 (26.5)
Age at informed consent, years	
*n*	268
Mean (SD)	10.7 (4.0)
95% CI	10.3, 11.2
Median	10.2
Min, max	3.7, 28.3
Age at first visit captured within the registry, years	
*n*	268
Mean (SD)	10.8 (4.0)
95% CI	10.3, 11.3
Median	10.2
Min, max	3.7, 28.3
Age at cutoff date, years	
*n*	268
Mean (SD)	14.8 (3.9)
95% CI	14.3, 15.3
Median	14.6
Min, max	6.1, 29.1
Age at ataluren start date, years	
*n*	268
Mean (SD)	9.8 (3.9)
95% CI	9.4, 10.3
Median	8.9
Min, max	5.0, 24.2
Total ataluren use duration, days	
* n*	268
Mean (SD)	1671 (566.8)
95% CI	1603, 1739
Median	1713
Min, max	133, 2736

*BMI* body mass index, *CI* confidence interval, *nmDMD* nonsense mutation Duchenne muscular dystrophy, *SD* standard deviation, *STRIDE* Strategic Targeting of Registries and International Database of Excellence

^a^Baseline data are data collected at first visit captured in the STRIDE Registry

^b^A minimum age value of zero was recorded owing to a prenatal diagnosis. Patients with a prenatal diagnosis recorded as the first symptom were excluded from the effectiveness analyses but were included in the evaluable population

#### Demographics of patients in the STRIDE Registry non-ambulatory population

Demographics and baseline characteristics of patients aged 5 years or older in the non-ambulatory population of the STRIDE Registry are shown in Supplementary Table 4. The mean (SD) age at first symptoms, and at genetic confirmation of nmDMD diagnosis was 2.9 (1.9) years and 6.1 (3.6) years, respectively. The mean (SD) age of the patients at the cut-off date was 17.2 (3.5) years.

#### Demographics of propensity-score matched STRIDE and CINRG DNHS patients

There were differences between the STRIDE effectiveness population (*n* = 261) and the CINRG DNHS population (*n* = 398) before propensity score matching. After propensity score matching, the CINRG DNHS population (*n* = 261) was more comparable to the STRIDE effectiveness population with respect to age at first symptoms, age at first corticosteroid use, duration of deflazacort use and duration of other corticosteroid use (Table 2 and Supplementary Table 5).

**Table 2:** Demographics and characteristics of patients in the STRIDE Registry and CINRG DNHS before and after propensity score matching

Demographic/characteristic	Unmatched population		Propensity-score matched population	
	STRIDE	CINRG	STRIDE	CINRG
	(*N* = 261)	(*N* = 398)	(*N* = 261)	(*N* = 261)
Age at first symptoms, years				
Mean (SD)	2.80 (1.66)	3.23 (1.68)	2.80 (1.66)	2.89 (1.41)
95% CI	2.60, 3.00	3.07, 3.40	2.60, 3.00	2.71, 3.06
Median	3.00	3.00	3.00	3.00
Min, max	0.1, 8.0	0.1, 8.0	0.1, 8.0	0.1, 8.0
* p* value	0.0012		0.5154	
Age at first corticosteroid use (excluding corticosteroid-naive patients)^a^, years	*n* = 233	*n* = 313	*n* = 233	*n* = 234
Mean (SD)	6.64 (2.17)	6.73 (2.05)	6.64 (2.17)	6.50 (1.94)
95% CI	6.36, 6.92	6.51, 6.96	6.36, 6.92	6.25, 6.75
Median	6.16	6.56	6.16	6.31
Min, max	2.9, 15.3	2.0, 14.3	2.9, 15.3	2.2, 13.9
* p* value	0.6059		0.4454	
Deflazacort use duration^c^, *n* (%)				
< 1 month or corticosteroid-naive	125 (47.9)	233 (58.5)	125 (47.9)	128 (49.0)
≥ 1 to < 12 months	11 (4.2)	20 (5.0)	11 (4.2)	13 (5.0)
≥ 12 months	125 (47.9)	145 (36.4)	125 (47.9)	120 (46.0)
* p* value	0.0138		0.8589	
Other corticosteroid duration^b^, *n* (%)				
< 1 month or corticosteroid-naive	141 (54.0)	203 (51.0)	141 (54.0)	131 (50.2)
≥ 1 to < 12 months	10 (3.8)	35 (8.8)	10 (3.8)	10 (3.8)
≥ 12 months	110 (42.1)	160 (40.2)	110 (42.1)	120 (46.0)
* p* value	0.0472		0.6695	
Total exposure to corticosteroids, days				
Mean (SD)	1838 (1403)	1285 (1238)	1838 (1403)	1580 (1238)
95% CI	1667, 2009	1163, 1407	1667, 2009	1429, 1731
Median	1641	1010	1641	1358
Min, max	0, 6566	0, 5970	0, 6566	0, 5970
* p* value	< 0.0001		0.0260	
Treatment follow-up duration, days				
Mean (SD)	1616 (581)	1653 (1025)	1616 (581)	1640 (1025)
95% CI	1545, 1687	1552, 1754	1545, 1687	1515, 1765
Median	1679	1792	1679	1655
Min, max	133, 2736	0, 3540	133, 2736	0, 3395
* p* value	0.5578		0.7488	

Propensity score model covariates include age at first symptoms, age at first corticosteroid use, duration of deflazacort use and duration of use of corticosteroids other than deflazacort

*CI* confidence interval, *CINRG DNHS* Cooperative International Neuromuscular Research Group Duchenne Natural History Study, *SD* standard deviation, *STRIDE* Strategic Targeting of Registries and International Database of Excellence

^a^Corticosteroid-naive patients were excluded to calculate the true age at first corticosteroid use. Corticosteroid-naive patients without age at first corticosteroid use data were captured by imputing their age at first corticosteroid use as 30 years; these data were imputed into the propensity-score calculations

^b^Corticosteroid duration is calculated from the date at which corticosteroid use was started to the date of loss of ambulation or the date of the last assessment. The same patient may be included in both the deflazacort duration and other corticosteroid duration groups if they had received both deflazacort and another corticosteroid at any one time

### Prior and concomitant medications

Of the 268 patients aged 5 years or older in the STRIDE Registry evaluable population, 261 (97.4%) are receiving medications that were started on or after the study entry date (Supplementary Table 6). These concomitant medications include corticosteroids (91.0%), vitamin D and vitamin D analog (81.0%), angiotensin-converting enzyme inhibitors (50.0%), calcium (25.4%), proton pump inhibitors (22.0%) and osmotically acting laxatives (10.1%).

### Ataluren use

The mean (SD) duration of ataluren use in the STRIDE Registry for patients aged 5 years or older in the evaluable population (*n* = 268) was 1671 (566.8) days (4.6 years), equivalent to 1226 patient years. The median (min, max) duration of ataluren exposure was 1713 (133, 2736). Compliance to ataluren (40 mg/kg/day) has been high; one patient stopped ataluren for 9 months and re-started at a lower dose and one patient had poor compliance.

### Safety of ataluren

#### TEAEs

TEAEs were reported for 131/284 patients (46.1%) aged 5 years or older, who experienced a total of 409 TEAEs (Table 3). Of the 131 patients who experienced TEAEs, 14 were not receiving corticosteroids. TEAEs were mostly mild (*n* = 45, 15.8%) or moderate (*n* = 51, 18.0%) and only 3.2% of them were considered related to ataluren. No TEAEs were life-threatening, and there were no deaths as of the data cut-off date. Eleven patients (4.2%) who used corticosteroids experienced TEAEs that led to discontinuation of ataluren. The most common TEAEs (in > 1% of patients) were gait inability (11.6% [33 patients]); fall (5.6% [16 patients]); headache (4.6% [13 patients]); back pain (3.9% [11 patients]); femur fracture (3.5% [10 patients]); off-label use and abdominal pain (3.2% [nine patients each]); ligament sprain (2.8% [eight patients]); vomiting (2.5% [seven patients]); constipation and cough (2.1% [6 patients each]); nasopharyngitis, upper abdominal pain, diarrhea, arthralgia, myalgia and cataracts (1.8% [five patients each]); humerus fracture, pyrexia, gastroenteritis, upper respiratory tract infection, epistaxis and hypertension (1.4% [four patients each]); and contusion, laceration, bronchitis, scoliosis, spinal fracture, myoglobinuria and obesity (1.1% [three patients each]) (Table 3). Twenty-seven patients (9.5%) experienced a total of 43 serious AEs (SAEs); of these, 23 patients used corticosteroids and four did not (Table 3). Most SAEs were determined by the investigator to be unrelated to ataluren except for left ventricular dysfunction, pneumonia and bronchitis; the relationship between these SAEs and ataluren could not be determined by the investigator. TEAEs in nine patients (3.2%) were considered related to ataluren; of these patients, eight used corticosteroids and one did not. TEAEs considered related to ataluren included upper abdominal pain (1.1% [three patients]); abdominal pain (0.7% [two patients]); and constipation, diarrhea, frequent bowel movements, vomiting, increased blood triglycerides, increased lipids and headache (0.4% [one patient each]). TEAEs in 19 patients (6.7%) were considered severe; of these patients, 16 used corticosteroids and three did not. TEAEs considered severe included gait inability (1.8% [five patients]); femur fracture (1.4% [four patients]); gastroenteritis and spinal fusion surgery (0.7% [two patients]); and tibia fracture, spinal fracture, fat embolism, peritonitis, arthralgia, ischemic stroke, weight decrease, acute respiratory failure, bronchial secretion retention, and osteosȳnthesis and tenotomy (0.4% [one patient each]). Safety data for patients aged ≥ 2 to < 5 years are shown in Supplementary Table 7.

**Table 3:** TEAEs experienced by patients aged 5 years or older in the as-treated population of the STRIDE Registry

	Corticosteroid use		All
	Yes	No	
	*N* = 259	*N* = 25	*N* = 284
Number of TEAEs^a^	364	45	409
Patients with at least one of the following, *n* (%)			
TEAE	117 (45.2)	14 (56.0)	131 (46.1)
TEAE related to ataluren	8 (3.1)	1 (4.0)	9 (3.2)
TEAE leading to discontinuation of ataluren	11 (4.2)	2 (8.0)	13 (4.6)
SAE	23 (8.9)	4 (16.0)	27 (9.5)
TEAE with maximum severity^b^			
Not reported	8 (3.1)	0 (0.0)	8 (2.8)
Unknown	6 (2.3)	2 (8.0)	8 (2.8)
Mild	41 (15.8)	4 (16.0)	45 (15.8)
Moderate	46 (17.8)	5 (20.0)	51 (18.0)
Severe	16 (6.2)	3 (12.0)	19 (6.7)
Life threatening	0 (0.0)	0 (0.0)	0 (0.0)
Patients with at least one of the following, *n* (%)^c,d^			
Injury, poisoning and procedural complications	51 (19.7)	5 (20.0)	56 (19.7)
Contusion	3 (1.2)	0 (0.0)	3 (1.1)
Fall	16 (6.2)	0 (0.0)	16 (5.6)
Femur fracture	9 (3.5)	1 (4.0)	10 (3.5)
Foot fracture	1 (0.4)	1 (4.0)	2 (0.7)
Humerus fracture	3 (1.2)	1 (4.0)	4 (1.4)
Laceration	3 (1.2)	0 (0.0)	3 (1.1)
Ligament sprain	8 (3.1)	0 (0.0)	8 (2.8)
Limb injury	2 (0.8)	0 (0.0)	2 (0.7)
Lip injury	2 (0.8)	0 (0.0)	2 (0.7)
Off-label use	7 (2.7)	2 (8.0)	9 (3.2)
Tibia fracture	2 (0.8)	0 (0.0)	2 (0.7)
Infections and infestations	26 (10.0)	5 (20.0)	31 (10.9)
Bronchitis	3 (1.2)	0 (0.0)	3 (1.1)
COVID-19	2 (0.8)	0 (0.0)	2 (0.7)
Gastroenteritis	4 (1.5)	0 (0.0)	4 (1.4)
Influenza	1 (0.4)	1 (4.0)	2 (0.7)
Nasopharyngitis	5 (1.9)	0 (0.0)	5 (1.8)
Otitis media	1 (0.4)	1 (4.0)	2 (0.7)
Rhinitis	2 (0.8)	0 (0.0)	2 (0.7)
Upper respiratory tract infection	4 (1.5)	0 (0.0)	4 (1.4)
General disorders and administration site conditions	33 (12.7)	5 (20.0)	38 (13.4)
Gait disturbance	2 (0.8)	0 (0.0)	2 (0.7)
Gait inability	28 (10.8)	5 (20.0)	33 (11.6)
Pyrexia	4 (1.5)	0 (0.0)	4 (1.4)
Gastrointestinal disorders	24 (9.3)	3 (12.0)	27 (9.5)
Abdominal pain	7 (2.7)	2 (8.0)	9 (3.2)
Upper abdominal pain	5 (1.9)	0 (0.0)	5 (1.8)
Constipation	6 (2.3)	0 (0.0)	6 (2.1)
Diarrhea	5 (1.9)	0 (0.0)	5 (1.8)
Nausea	1 (0.4)	1 (4.0)	2 (0.7)
Vomiting	6 (2.3)	1 (4.0)	7 (2.5)
Musculoskeletal and connective tissue disorders	25 (9.7)	2 (8.0)	27 (9.5)
Arthralgia	3 (1.2)	2 (8.0)	5 (1.8)
Back pain	11 (4.2)	0 (0.0)	11 (3.9)
Muscle spasms	2 (0.8)	0 (0.0)	2 (0.7)
Musculoskeletal pain	2 (0.8)	0 (0.0)	2 (0.7)
Myalgia	5 (1.9)	0 (0.0)	5 (1.8)
Pain in extremity	2 (0.8)	0 (0.0)	2 (0.7)
Scoliosis	3 (1.2)	0 (0.0)	3 (1.1)
Spinal fracture	3 (1.2)	0 (0.0)	3 (1.1)
Psychiatric disorders	9 (3.5)	0 (0.0)	9 (3.2)
Autism spectrum disorder	2 (0.8)	0 (0.0)	2 (0.7)
Initial insomnia	2 (0.8)	0 (0.0)	2 (0.7)
Sleep disorder	2 (0.8)	0 (0.0)	2 (0.7)
Respiratory, thoracic and mediastinal disorders	11 (4.2)	3 (12.0)	14 (4.9)
Cough	6 (2.3)	0 (0.0)	6 (2.1)
Epistaxis	2 (0.8)	2 (8.0)	4 (1.4)
Nervous system disorders	16 (6.2)	1 (4.0)	17 (6.0)
Headache	12 (4.6)	1 (4.0)	13 (4.6)
Migraine	2 (0.8)	0 (0.0)	2 (0.7)
Investigations	14 (5.4)	2 (8.0)	16 (5.6)
Blood triglycerides increased	2 (0.8)	0 (0.0)	2 (0.7)
Body height below normal	2 (0.8)	0 (0.0)	2 (0.7)
Lipids increased	2 (0.8)	0 (0.0)	2 (0.7)
Surgical and medical procedures	8 (3.1)	1 (4.0)	9 (3.2)
Spinal fusion surgery	2 (0.8)	0 (0.0)	2 (0.7)
Eye disorders	7 (2.7)	0 (0.0)	7 (2.5)
Cataracts	5 (1.9)	0 (0.0)	5 (1.8)
Renal and urinary disorders	7 (2.7)	0 (0.0)	7 (2.5)
Myoglobinuria	3 (1.2)	0 (0.0)	3 (1.1)
Pollakiuria	2 (0.8)	0 (0.0)	2 (0.7)
Skin and subcutaneous tissue disorders	7 (2.7)	0 (0.0)	7 (2.5)
Rash	2 (0.8)	0 (0.0)	2 (0.7)
Cardiac disorders	6 (2.3)	0 (0.0)	6 (2.1)
Left ventricular dysfunction	2 (0.8)	0 (0.0)	2 (0.7)
Tachycardia	2 (0.8)	0 (0.0)	2 (0.7)
Metabolism and nutrition disorders	5 (1.9)	0 (0.0)	5 (1.8)
Obesity	3 (1.2)	0 (0.0)	3 (1.1)
Vascular disorders	5 (1.9)	0 (0.0)	5 (1.8)
Hypertension	4 (1.5)	0 (0.0)	4 (1.4)
Hepatobiliary disorders	3 (1.2)	1 (4.0)	4 (1.4)
Endocrine disorders	3 (1.2)	0 (0.0)	3 (1.1)
Delayed puberty	2 (0.8)	0 (0.0)	2 (0.7)
Ear and labyrinth disorders	2 (0.8)	0 (0.0)	2 (0.7)

*AE* adverse event, *COVID-19* coronavirus disease 2019, *STRIDE* Strategic Targeting of Registries and International Database of Excellence, *TEAE* treatment-emergent adverse event, *SAE* serious adverse event

^a^TEAE is defined as any AE with a start date on or after the first date of ataluren use or worsened after initiation of ataluren and does not necessarily have a causal relationship with ataluren. Events with missing severity are not reported

^b^For patients with two or more TEAEs, the event with the maximum severity was reported. The order of severity is ‘not reported’, ‘unknown’, ‘mild’, ‘moderate’, ‘severe’ and ‘life threatening’

^c^AEs were coded using the Medical Dictionary for Regulatory Activities (version 20.1)

^d^A patient who reported at least one occurrence with the same preferred term was counted only once for that term

#### Laboratory results

Laboratory results including serum lipid profiles, blood pressure, hepatic and renal tests and cardiac measurements are shown in Supplementary Tables 8–12. Equivalent data for patients aged ≥ 2 to < 5 years are shown in Supplementary Tables 13–17. Clinically significant laboratory abnormalities were infrequent, and no clinically meaningful trends were observed in these laboratory assessments. Shifts in results from normal at the time of the first assessment to clinically significantly abnormal were infrequent for high-density lipoprotein, low-density lipoprotein, triglycerides, and total cholesterol (Supplementary Table 8). Corticosteroids can act as confounding factors on lipid assessments. Therefore, shifts in serum lipid results are reported for STRIDE patients who did not receive corticosteroids (Supplementary Table 9). One patient (6.7%) had a shift in total cholesterol from normal at first assessment to clinically significantly abnormal. Of the 190 patients (66.9%) with at least two ECG assessments, four patients (2.1%) experienced shifts in ECG results from normal at first assessment to clinically significantly abnormal. These four patients used corticosteroids. Of 184 patients (64.8%) with at least two heart rhythm assessments, 32 patients (17.4%) experienced shifts in results from normal at first assessment to abnormal. Thirty of these patients used corticosteroids (Supplementary Table 10).

Of the 139 patients (59.1%) receiving corticosteroids who had normal blood pressure values at the time of first assessment, 21 patients (8.9%) experienced a shift in values from normal to prehypertensive and 65 patients (27.7%) experienced a shift in values from normal to hypertensive. Of the 13 patients (76.5%) not receiving corticosteroids who had normal blood pressure values at the time of first assessment, two patients (11.8%) experienced a shift in values from normal to prehypertensive and six patients (35.5%) experienced a shift in values from normal to hypertensive (Supplementary Table 11). Shifts in pulse rate from normal at first assessment to elevated or low rates were infrequent (Supplementary Table 11).

There were no shifts from normal at the time of first assessment to clinically significantly abnormal in levels of the hepatic enzymes aspartate aminotransferase and alanine transaminase or in renal test results for blood urea nitrogen (Supplementary Table 12). Test results for gamma-glutamyl transferase levels and total bilirubin hepatic enzyme levels showed that one patient in each analysis shifted from normal at the time of first assessment to clinically significantly abnormal. One patient with normal cystatin C levels at the time of first assessment shifted to clinically significantly abnormal. As expected for individuals with DMD, many patients had abnormally low levels of serum creatinine at first assessment and throughout the study; three patients shifted from normal at the time of first assessment to clinically significantly abnormal [19].

### Effectiveness of ataluren

#### Age at loss of ambulation

**Fig. 2:**
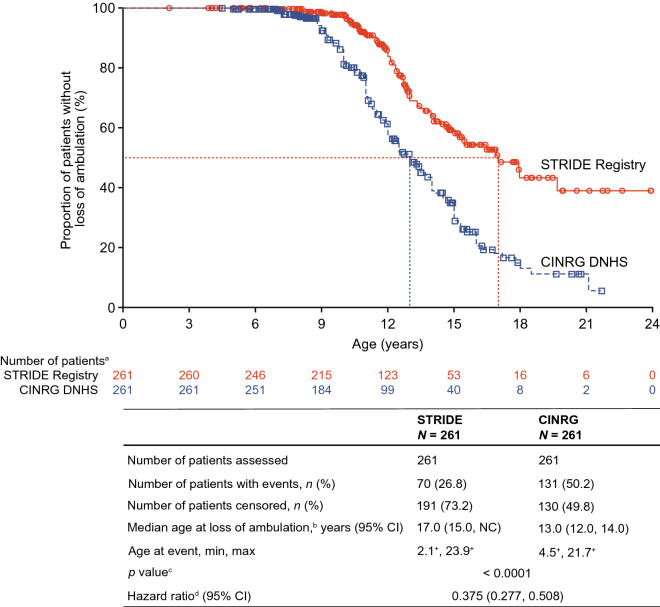
Age at loss of ambulation^b^ for propensity-score matched patients from the STRIDE Registry and CINRG DNHS. Propensity-score model covariates include age at first symptoms, age at first corticosteroid use, duration of deflazacort use and duration of use of corticosteroids other than deflazacort. Censor dates for censored patients in the STRIDE Registry were derived from the last assessment date across treatment, physical examination, vital signs, 6-min walk distance, timed function tests, North Star Ambulatory Assessments, percentage of predicted FVC and upper limb function tests. ‘+’ indicates a censored observation. Corticosteroid duration is calculated from the date at which corticosteroid use was started to the date of loss of ambulation or the date of the last assessment. *CINRG DNHS* Cooperative International Neuromuscular Research Group Duchenne Natural History Study, *NC* not calculable, *STRIDE* Strategic Targeting of Registries and International Database of Excellence. ^a^Number of patients at risk of having the event (loss of ambulation). ^b^Loss of ambulation was defined as full-time wheelchair use. ^c^*p* value is from a log-rank test stratified by deflazacort and other corticosteroid usage durations. ^d^Hazard ratio is from stratified (by durations of deflazacort and other corticosteroid use), Cox regression with study, age at first symptoms and age at first corticosteroid use as covariates. The hazard ratio is STRIDE Registry versus CINRG DNHS

Of the 261 patients in the STRIDE Registry effectiveness population, 70 (26.8%) lost ambulation; of the 261 propensity-score matched patients in the CINRG DNHS, 131 (50.2%) lost ambulation. The median (95% CI) ages at loss of ambulation for the matched STRIDE Registry and CINRG DNHS populations were 17.0 years (15.0, NC) and 13.0 years (12.0, 14.0), respectively (*p* < 0.0001) (Fig. 2).

#### Age at decline in pulmonary function

**Fig. 3:**
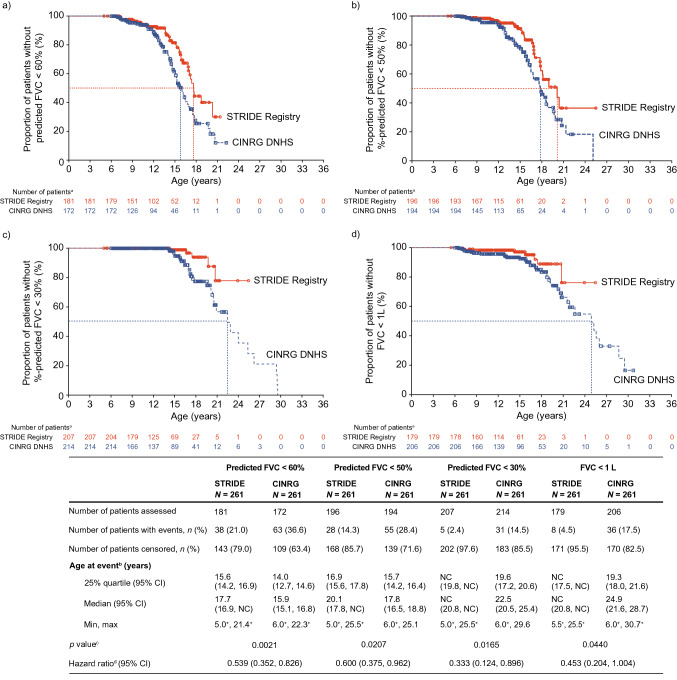
Age at predicted FVC of **a** < 60%, **b** < 50% and **c** < 30%, and at **d** FVC < 1 L for propensity-score matched patients from the STRIDE Registry and CINRG DNHS. Propensity-score model covariates include age at first symptoms, age at first corticosteroid use, duration of deflazacort use and duration of use of corticosteroids other than deflazacort. Censor dates for censored patients in the STRIDE Registry were derived from the last assessment date across treatment, physical examination, vital signs, 6-min walk distance, timed function tests, North Star Ambulatory Assessments, percentage of predicted FVC and upper limb function tests. ‘+’ indicates a censored observation. Corticosteroid duration is calculated from the date at which corticosteroid use was started to the date of the last assessment. The hazard ratio is STRIDE Registry versus CINRG DNHS. *CINRG DNHS* Cooperative International Neuromuscular Research Group Duchenne Natural History Study, *FVC* forced vital capacity, *NC* not calculable, *STRIDE* Strategic Targeting of Registries and International Database of Excellence. ^a^Number of patients at risk of having the event. ^b^Event = %-predicted FVC < 60%, < 50% or < 30% or FVC < 1 L. ^c^*p* value is from a log-rank test stratified by deflazacort and other corticosteroid usage durations. ^d^Hazard ratio is from stratified (by durations of deflazacort and other corticosteroid use) Cox regression with study, age at first symptoms and age at first corticosteroid use as covariates

Of the patients in the propensity-score matched STRIDE Registry and CINRG DNHS populations, patients declined to a median (95% CI) age at predicted FVC < 60% at 17.7 (16.9, NC) years and 15.9 (15.1, 16.8) years (*p* = 0.0021), respectively (Fig. 3a). Patients declined to a median (95% CI) age at predicted FVC < 50% at 20.1 (17.8, NC) years in the STRIDE Registry and 17.8 (16.5, 18.8) years in the CINRG DNHS (*p* = 0.0207) (Fig. 3b). The median age at decline to predicted FVC < 30% and FVC < 1 L were not estimable for STRIDE patients; CINRG DNHS patients declined to these thresholds at a median (95% CI) age of 22.5 (20.5, 25.4) years and 24.9 (21.6, 28.7) years, respectively (Fig. 3c, d).

#### Annual rate of predicted FVC decline for non-ambulatory patients

**Fig. 4:**
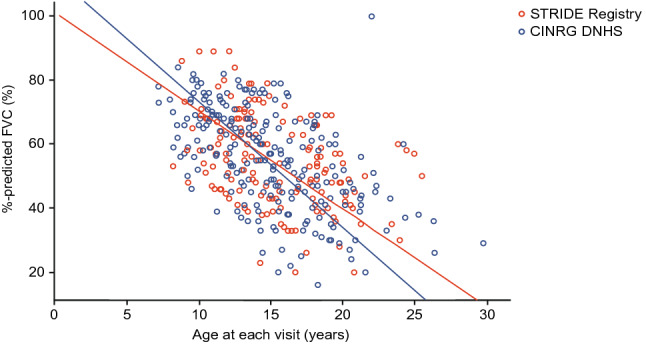
Annual rate of predicted FVC decline for propensity-score matched STRIDE Registry and CINRG DNHS non-ambulatory patients^a^ with a baseline-predicted FVC ≥ 30% and ≤ 80% at first assessment. To be eligible for propensity score matching in these analyses, patients from both the STRIDE Registry and CINRG DNHS must have been non-ambulatory at their last assessment (but can be ambulatory or non-ambulatory at first assessment), the duration between their first and last assessments must be ≥ 48 weeks and they must have a predicted FVC ≥ 30% and ≤ 80% at first assessment. STRIDE patients must have received ataluren for ≥ 12 months on their first assessment. Propensity score matching was performed to identify non-ambulatory CINRG DNHS patients who were comparable to STRIDE patients in the effectiveness population using the following five covariates: age at first symptom onset; age at first corticosteroid use; duration of deflazacort use to the last assessment (i.e., with a predicted FVC ≥ 30%); duration of other steroid use to the last assessment (i.e., with a predicted FVC ≥ 30%); and time from loss of ambulation to the last assessment. *CINRG DNHS* Cooperative International Neuromuscular Research Group Duchenne Natural History Study, *FVC* forced vital capacity, *STRIDE* Strategic Targeting of Registries and International Database of Excellence. ^a^The non-ambulatory STRIDE population is defined as the subset of the effectiveness population who were full-time wheelchair users or bedridden on or before first recorded commercial or early-access program ataluren use or anytime during the study

The annual change (95% CI) in predicted FVC decline for non-ambulatory patients with a baseline predicted FVC ≥ 30% and ≤ 80% at first assessment was − 3.07 (− 3.92, − 2.21) and − 3.95 (− 4.50, − 3.39) in the STRIDE Registry and CINRG DNHS, respectively (*p* = 0.0854) (Fig. 4). Median (95% CI) ages at loss of ambulation for these STRIDE and CINRG DNHS non-ambulatory patients were 12.1 (11.5, 12.7) years and 11.5 (10.4, 13.4) years, respectively (*p* = 0.9944). Median (95% CI) ages at decline to a predicted FVC of less than 50% were 18.2 (15.1, 20.4) years and 17.8 (14.4, NA) years, respectively (*p* = 0.3354).

#### Change in BMI

Baseline BMI by age at consent date can be found in Supplementary Fig. 3. Of the propensity-score matched STRIDE Registry and CINRG DNHS populations, 11/211 patients (5.2%) and 10/167 patients (6.0%), respectively, experienced a shift in BMI from normal to obese between their first and extreme assessment (Supplementary Table 18). BMI *z*-scores for propensity-score matched STRIDE Registry and CINRG DNHS populations can be found in Supplementary Fig. 4.

## Discussion

Since the first STRIDE Registry data cut-off on July 9, 2018 [9, 10], the registry population and number of participating countries and study sites have increased, providing a more statistically meaningful sample size and diverse population than was previously analyzed. The total number of patients enrolled has increased from 217 to 307. Three additional countries (Brazil, Latvia, and Portugal) are now actively recruiting patients, increasing the number of participating countries to 14, and there are currently 66 active study sites, an increase of 13. Owing to a protocol amendment, 22 patients aged ≥ 2 to < 5 years have been included in STRIDE since the 2018 data cut-off date. Given that this subpopulation is relatively small, patients aged 5 years or older were the focus in this publication.

Since the 2018 data cut-off date*,* the mean duration of ataluren exposure has increased substantially. For patients aged 5 years or older (*n* = 268), ataluren was used for a mean duration of 1671 days (4.6 years), equivalent to 1226 patient-years. This is an increase of 1032 days (2.8 years) and 853.4 patient-years of ataluren exposure since the 2018 data cut-off [9], meaning that the long-term safety and effectiveness of ataluren can be more accurately assessed.

As of the latest data cut-off of January 31, 2022, the mean age at genetic diagnosis of nmDMD was 5.4 years, similar to that reported at the 2018 data cut-off date (i.e., 5.2 years). As previously discussed [9], the later age at genetic diagnosis of individuals with nmDMD in the STRIDE Registry compared with previous European studies [20, 21] is likely due to the delays caused by the longer sequential genetic diagnostic process for these patients; comparisons cannot be accurately made between these studies owing to the different genetic mutations, demographics of the populations and differences in genetic test completion [9]. Despite this, more needs to be done to reduce the time to genetic diagnosis of all individuals with DMD.

Ataluren was well tolerated and its safety profile and laboratory test results were consistent with those shown in previous clinical trials of ataluren and at the 2018 STRIDE Registry data cut-off [9, 10, 22–24]. As previously reported [10], some patients were excluded from the as-treated population owing to mistakenly being diagnosed with nonsense mutation DMD, and 'off-label' use was included as a TEAE for some patients in this data cut. It should be noted that since the initiation of the STRIDE Registry, there have been improvements in the monitoring of incorrect genetic diagnoses of nmDMD patients and usage of ataluren. For example, in the UK, it is common practice to ensure that every patient with nmDMD referred for ataluren treatment is first vetted by a national panel which includes geneticists and neuromuscular specialists.

As of the latest STRIDE Registry data cut-off date of January 31, 2022, STRIDE patients receiving ataluren plus SoC showed a significant 4-year delay in the median age at loss of ambulation compared with propensity-score matched CINRG DNHS patients receiving SoC alone (17.0 years vs 13.0 years, respectively; *p* < 0.0001). At the 2018 data cut-off, STRIDE patients had a delay in median age at loss of ambulation of 3.5 years (14.5 years vs 11.0 years; *p* < 0.0001). Given the additional 2.8 years of ataluren exposure, the 2022 data may show that ataluren confers a cumulative benefit to individuals with nmDMD. These data add to the body of evidence indicating that ataluren delays loss of ambulation in individuals with DMD. Study 019, a long-term Phase 3 study of ataluren in patients with a history of ataluren exposure showed a 2.2-year delay in loss of ambulation compared with patients receiving SoC alone [24]. It is worth noting that STRIDE patients required no specific duration of exposure to ataluren making the analyses of a treatment effect more conservative.

Results from the 2018 data cut-off *suggested* a trend toward a delay in worsening of pulmonary function for patients in STRIDE compared with those in CINRG DNHS, as measured by reductions in %-predicted FVC of less than 60% and 50% [10]. However, owing to the relatively short follow-up of patients in the STRIDE Registry at this data cut-off and low number of patients reaching these milestones at that stage, it was not possible to draw firm conclusions. As of the January 31, 2022, data cut-off, results for decline in pulmonary function showed that ataluren plus SoC delayed age at predicted FVC of less than 60% by 1.8 years (*p* = 0.0021) and delayed predicted FVC of less than 50% by 2.3 years (*p* = 0.0207), suggesting ataluren delays decline in pulmonary function. Predicted FVC of less than 30% was not analyzed for the previous data cut-off, given that the STRIDE patients would not have yet declined to this threshold [10]. Patients with a predicted FVC of less than 30% are considered to have severe respiratory insufficiency and require ventilation [17, 24, 25]. As of the latest data cut-off, patients in the STRIDE Registry had still not yet declined to a predicted FVC of less than 30% and FVC of less than 1 L, whereas CINRG DNHS patients reached these FVC milestones at median ages of 22.5 years and 24.9 years, respectively. Therefore, results for these milestones will be reviewed at a later data cut-off.

The annual change in predicted FVC decline for non-ambulatory patients was − 3.07 and − 3.95 in the STRIDE Registry and CINRG DNHS, respectively (*p* = 0.0854). These analyses were not performed at the 2018 data cut-off; they aimed to assess whether there was a difference in the annual decline in FVC in patients who were at a linear rate of decline (i.e., patients with a predicted FVC < 30% and FVC < 1 L were excluded from these analyses). An annual change in predicted FVC of – 3.95 is less than previously reported for the natural history of DMD [26]; the results presented here suggest that the rate of pulmonary decline between the STRIDE Registry patients and CINRG DNHS patients is similar; however, the rate of decline was numerically smaller in the STRIDE population but the small sample size limits the interpretation of these data.

Although the annual rate of predicted FVC decline for matched STRIDE Registry and CINRG DNHS non-ambulatory patients was not statistically different, the results trend toward ataluren delaying decline in pulmonary function. An increase in the sample size may result in a more statistically meaningful analysis.

Of the propensity-score matched STRIDE Registry and CINRG DNHS populations, 11/211 STRIDE patients (5.2%) and 10/167 CINRG DNHS patients (6.0%) experienced a shift in BMI from normal to obese between their first and extreme assessment. This shows that ataluren plus SoC is not associated with changes in BMI compared with SoC alone and indirectly suggests that data matching for steroid exposure is effective.

Although randomized control trials are considered the gold standard when determining the effectiveness of a treatment, the collection of real-world evidence (RWE) reflects the clinical experience of a more heterogeneous and larger population. It is well-documented that the collection of RWE has several limitations; data may be inconsistently captured, or the recording of key endpoints may be missed. For studies without an internal comparator, selection bias must be considered. Individuals in STRIDE, the only observational study of its kind including exclusively individuals with nmDMD, could not be compared to a like-for-like population, and were therefore compared with participants in the CINRG DNHS, a general DMD population. The use of propensity score matching reduced selection bias for known confounders (it cannot compensate for unknown confounders); the covariates used here for propensity score matching were selected following an extensive literature search on factors which impact DMD progression and consequently allow the populations to be compared and ataluren effectiveness to be assessed. Although the covariates would ideally have included baseline functional data, given that baseline motor performance is associated with age at loss of ambulation [27], these were not completely available for both STRIDE and CINRG DNHS populations.

Additionally, owing to the non-existence of a comparator population including a correspondingly large number of patients with nmDMD, a limitation of these analyses is that the STRIDE and CINRG DNHS patients were not matched based on mutation type or location, which have been reported to affect DMD progression [28–30]. Therefore, the best possible alternatives of the most routinely captured disease progression predictor data were used as covariates. It is also worth noting that although mutation type can affect DMD progression, Bello et al*.* reported that patients in the CINRG DNHS with nmDMD had a similar disease severity to patients with other DMD mutations, as demonstrated by a similar age at loss of ambulation [28]. Moreover, while the location of a DMD mutation can also affect the rate of motor decline in DMD owing to the different dystrophin isoforms produced [31], this impact can be negated with appropriate clinical matching [32].

In a recent study, it was shown that a daily prednisone or deflazacort corticosteroid regimen is more effective at delaying DMD progression than an intermittent prednisone regimen [33]. It would, therefore, have been beneficial to use corticosteroid regimen as a covariate for propensity score matching. However, patients in the STRIDE Registry may have changed their corticosteroid regimen during their participation, which would reduce the number of patients who could be included in discrete corticosteroid regimen categories, and therefore reduce the number of patients available for matching and the sample size of the effectiveness analyses.

The STRIDE Registry is the first drug registry for DMD and is demonstrating the safety and effectiveness of ataluren over a long treatment exposure in routine clinical practice. The next interim analysis of STRIDE data will be in 2023; the final data cut-off date will be in 2025. Future data cut-offs including individuals with even more prolonged ataluren exposure will continue to assess the long-term safety and effectiveness of ataluren and will shed further light on the evolving course of DMD disease progression in ataluren-treated individuals.

## Conclusion

The STRIDE Registry has grown since its first data cut-off in 2018 and its data have matured, with an additional 853.4 patient-years of ataluren exposure logged. Results from this 2022 data cut-off show that long-term ataluren use (4.6 years) in real-world clinical practice is associated with favorable safety data and delays in several disease progression milestones.

## Supplementary Information

Below is the link to the electronic supplementary material.

Supplementary file1 (PDF 1043 KB)

## Data Availability

The authors certify that this manuscript reports original real-world evidence data (NCT02369731). Individual patient data will not be made publicly available. The study protocol is available indefinitely to interested parties on request.
