# Correction to: Safety and effectiveness of ataluren in patients with nonsense mutation DMD in the STRIDE Registry compared with the CINRG Duchenne Natural History Study (2015–2022): 2022 interim analysis

**DOI:** 10.1007/s00415-023-11864-2

**Published:** 2023-07-17

**Authors:** Eugenio Mercuri, Andrés Nascimento Osorio, Francesco Muntoni, Filippo Buccella, Isabelle Desguerre, Janbernd Kirschner, Már Tulinius, Maria Bernadete Dutra de Resende, Lauren P. Morgenroth, Heather Gordish-Dressman, Shelley Johnson, Allan Kristensen, Christian Werner, Panayiota Trifillis, Erik K. Henricson, Craig M. McDonald, the STRIDE and CINRG DNHS investigators

**Affiliations:** 1 Department of Pediatric Neurology, Catholic University, Rome, Italy; 2 Centro Clinico Nemo, Fondazione Policlinico Agostino Gemelli IRCCS, Rome, Italy; 3 Neuromuscular Unit, Department of Neurology and Research in Neuromuscular Diseases, Institut de Recerca Sant Joan de Déu, Center for Biomedical Research Network on Rare Diseases (CIBERER), ISCIII, Barcelona, Spain; 4 UCL Great Ormond Street Institute of Child Health, London, UK; 5 National Institute for Health Research, Great Ormond Street Institute of Child Health Biomedical Research Centre, University College London, London, UK; 6 Parent Project APS, Rome, Italy; 7 Hôpital Necker-Enfants Malades, Paris, France; 8 Department of Neuropediatrics and Muscle Disorders, Medical Center-University of Freiburg, Faculty of Medicine, Freiburg, Germany; 9 Department of Pediatrics, Gothenburg University, Queen Silvia Children’s Hospital, Gothenburg, Sweden; 10 Department of Neurology, Faculty of Medicine, University of São Paulo, São Paulo, SP Brazil; 11 Therapeutic Research in Neuromuscular Disorders Solutions (TRiNDS), Pittsburgh, PA USA; 12 Center for Genetic Medicine, Children’s National Health System and the George Washington, Washington, DC USA; 13 PTC Therapeutics Inc., South Plainfield, NJ USA; 14 PTC Therapeutics Germany GmbH, Frankfurt, Germany; 15 University of California Davis School of Medicine, Davis, CA USA

**Correction to: Journal of Neurology** 10.1007/s00415-023-11687-1

The original version of this article unfortunately contained a mistake. There are 2 data errors in the results section of the abstract.

In abstract, first sentence of the results section should read as

As of January 31, 2022, 307 patients were enrolled from 14 countries. Mean (standard deviation [SD]) ages at first symptoms and at genetic diagnosis were 2.9 (1.7) years and 5.4 (3.1) years, respectively. Mean (SD) duration of ataluren exposure was 1671 (566.8) days.

