# Supplementary material for: Safety and effectiveness of ataluren in patients with nonsense mutation DMD in the STRIDE Registry compared with the CINRG Duchenne Natural History Study (2015–2022): 2022 interim analysis
Source: J Neurol. 2023 Apr 28;270(8):3896–913. doi: 10.1007/s00415-023-11687-1 (PMC10141820; doi:10.1007/s00415-023-11687-1)
Supplement: Supplementary file 1 — Supplementary file1 (PDF 1043 KB) [file 415_2023_11687_MOESM1_ESM.pdf]

Supplementary materials

Title: Safety and effectiveness of ataluren in patients with nonsense mutation DMD in the STRIDE Registry compared with the CINRG Duchenne Natural History Study (2015–2022): 2022 interim analysis

Authors: Eugenio Mercuri[1,2] · Andrés Nascimento Osorio[3] · Francesco Muntoni[4,5] · Filippo Buccella[6] · Isabelle Desguerre[7] · Janbernd Kirschner[8] · Már Tulinius[9] · Maria Bernadete Dutra de Resende[10] · Lauren P Morgenroth[11] · Heather Gordish-Dressman[12] · Shelley Johnson[13] · Allan Kristensen[13] · Christian Werner[14] · Panayiota Trifillis[13] · Erik K Henricson[15] · Craig M McDonald[15] on behalf of the STRIDE and CINRG DNHS investigators

Affiliations:

[1]Department of Pediatric Neurology, Catholic University, Rome, Italy

[2]Centro Clinico Nemo, Fondazione Policlinico Agostino Gemelli IRCCS, Rome Italy

[3]Neuromuscular Unit, Department of Neurology, and Research in Neuromuscular Diseases, Institut de Recerca I Sant Joan de Déu, Center for Biomedical Research Network on Rare Diseases (CIBERER), ISCIII, Spain

[4]UCL Great Ormond Street Institute of Child Health, London, UK

[5]National Institute for Health Research, Great Ormond Street Institute of Child Health Biomedical Research Centre, University College London, London, UK

[6]Parent Project APS, Rome, Italy

[7]Hôpital Necker – Enfants Malades, Paris, France

[8]Department of Neuropediatrics and Muscle Disorders, Medical Center – University of Freiburg, Faculty of Medicine, Freiburg, Germany

[9]Department of Pediatrics, Gothenburg University, Queen Silvia Children's Hospital, Gothenburg, Sweden

[10]Department of Neurology, Faculty of Medicine, University of São Paulo, São Paulo SP, Brazil

[11]Therapeutic Research in Neuromuscular Disorders Solutions (TRiNDS), Pittsburgh, PA, USA

[12]Center for Genetic Medicine, Children's National Health System and the George Washington, Washington, DC, USA

[13]PTC Therapeutics Inc., South Plainfield, NJ, USA

[14]PTC Therapeutics Germany GmbH, Frankfurt, Germany

[15]University of California Davis School of Medicine, Davis, CA, USA

Corresponding author and email address: Eugenio Mercuri;
eugeniomaria.mercuri@unicatt.it

Supplementary Table 1 Number of patients enrolled in STRIDE by country

		**Patients enrolled as of March 31, 2022** ***N* = 307**
**Country**	Number of sites	Number of patients
Austria	2	4
Brazil	1	15
Czech Republic	2	10
France	17	56
Germany	9	34
Greece	2	6
Hungary	1	2
Israel	3	8
Italy	12	67
Latvia	1	2
Portugal	2	8
Romania	2	14
Sweden	2	13
UK	10	68

STRIDE Strategic Targeting of Registries and International Database of Excellence

Supplementary Table 2 Patient race in the STRIDE Registry evaluable population

Race, *n* (%)	All patients<br>*N* = 290
White	213 (73.4)
Arab/Middle Eastern	8 (2.8)
Arab/Middle Eastern, Asian	1 (0.3)
Asian	6 (2.1)
Black	3 (1.0)
Mixed race, black/white	1 (0.3)
North African	1 (0.3)
Latin	1 (0.3)
Unknown	4 (1.4)

STRIDE Strategic Targeting of Registries and International Database of Excellence

Supplementary Table 3 Demographics and characteristics of patients aged ≥ 2 to < 5 years in the STRIDE Registry evaluable population

	**All patients** *N* **= 22**
Baseline[a] weight, kg	
n	17
Mean (SD)	16.3 (2.4)
95% CI	15.0, 17.5
Median	16.5
Min, max	11.8, 20.0
Baseline[a] height, cm	
n	16
Mean (SD)	99.1 (7.8)
95% CI	94.9, 103.3
Median	99.1
Min, max	84.0, 111.0
Baseline[a] BMI, kg/m^2	
n	15
Mean (SD)	16.1 (1.4)
95% CI	15.3, 16.9
Median	16.1
Min, max	13.7, 18.7
Age at first symptoms, years	
n	20
Mean (SD)	1.7 (1.1)
95% CI	1.2, 2.2
Median	1.8
Min, max	0.3, 4.0
Age at muscle biopsy, years	
n	5
Mean (SD)	2.3 (0.9)
95% CI	1.1, 3.4
Median	2.8
Min, max	0.9, 3.0
Age at genetic confirmation of nmDMD diagnosis, years	
n	22
Mean (SD)	1.9 (0.9)
95% CI	1.5, 2.4
Median	1.65
Min, max	0.4, 3.5
Previously enrolled in ataluren clinical trial, *n* (%)	
No	22 (100.0)
Yes	0 (0.0)
Age at informed consent, years	
n	22
Mean (SD)	4.1 (1.1)
95% CI	3.6, 4.6
Median	4.0
Min, max	2.1, 6.7

Age at first visit captured within the registry, years	
n	22
Mean (SD)	4.1 (1.2)
95% CI	3.6, 4.6
Median	4.0
Min, max	2.1, 6.7
Age at cutoff date, years	
n	22
Mean (SD)	6.6 (1.7)
95% CI	5.8, 7.4
Median	6.2
Min, max	4.1, 10.5
Age at ataluren start date, years	
n	22
Mean (SD)	3.8 (0.9)
95% CI	3.4, 4.2
Median	3.9
Min, max	2.1, 5.0
Ataluren use duration, days	
n	22
Mean (SD)	1034 (472.2)
95% CI	824.7, 1234
Median	926.0
Min, max	351, 2238

BMI body mass index, *CI* confidence interval, *nmDMD* nonsense mutation Duchenne muscular dystrophy, *SD* standard deviation, *STRIDE* Strategic Targeting of Registries and International Database of Excellence

[a]Baseline data are data collected at the first visit captured in the STRIDE Registry

Supplementary Table 4 Demographics and characteristics of patients aged 5 years or older in the STRIDE Registry non-ambulatory population

	**All patients** **_N_ = 81**
Baseline[a] weight, kg	
n	70
Mean (SD)	36.6 (15.2)
95% CI	33.0, 40.2
Median	35.3
Min, max	13.5, 78.0
Baseline[a] height, cm	
n	53
Mean (SD)	130.7 (15.9)
95% CI	126.3, 135.1
Median	128.0
Min, max	94.3, 168.0
Baseline[a] BMI, kg/m^2	
n	53
Mean (SD)	20.8 (5.7)
95% CI	19.2, 22.4
Median	19.3
Min, max	13.9, 40.1
Age at first symptoms, years	
n	76
Mean (SD)	2.9 (1.9)
95% CI	2.5, 3.3
Median	3.0
Min, max	0.1, 8.0
Age at muscle biopsy, years	
n	59
Mean (SD)	5.0 (2.3)
95% CI	4.4, 5.6
Median	4.6
Min, max	1.2, 11.1
Age at genetic confirmation of nmDMD diagnosis, years	
n	77
Mean (SD)	6.1 (3.6)
95% CI	5.3, 6.9
Median	5.6
Min, max	0.02, 23.0
Previously enrolled in ataluren clinical trial, *n* (%)	
No	46 (56.8)
Yes	35 (43.2)
Age at informed consent, years	
n	81
Mean (SD)	12.8 (3.8)
95% CI	12.0, 13.7
Median	12.1
Min, max	5.3, 28.3

Age at first visit captured within the registry, years	
n	81
Mean (SD)	12.9 (3.8)
95% CI	12.0, 13.7
Median	12.1
Min, max	5.3, 28.3
Age at cutoff date, years	
n	81
Mean (SD)	17.2 (3.5)
95% CI	16.4, 18.0
Median	17.1
Min, max	10.1, 29.1
Age at ataluren start date, years	
n	81
Mean (SD)	11.7 (4.1)
95% CI	10.8, 12.6
Median	10.8
Min, max	5.3, 24.2

BMI body mass index, *CI* confidence interval, *nmDMD* nonsense mutation Duchenne muscular dystrophy, *SD* standard deviation, *STRIDE* Strategic Targeting of Registries and International Database of Excellence

[a]Baseline data are data collected at the first visit captured in the STRIDE Registry

Supplementary Table 5 Additional demographics and characteristics for the propensity-score matched STRIDE Registry and CINRG DNHS populations

	Propensity-matched population	
**Demographic/characteristic**	**STRIDE (*N* = 261)**	**CINRG (*N* = 261)**
**Age at first assessment, years**		
Mean (SD)	9.3 (4.0)	10.1 (5.3)
SE	0.2	0.3
95% CI	8.8, 9.7	9.5, 10.8
Median	8.5	8.8
Min, max	2.1, 23.6	2.1, 28.0
p value	0.0313	
**Age at last assessment, years**		
Mean (SD)	13.1 (4.4)	14.6 (6.3)
SE	0.3	0.4
95% CI	12.6, 13.6	13.9, 15.4
Median	12.9	14.4
Min, max	2.1, 25.5	4.5, 33.1
p value	0.0012	
**Any steroid use duration, *n* (%)**		
< 1 month or corticosteroid-naive	30 (11.5)	28 (10.7)
≥ 1 to < 12 months	13 (5.0)	17 (6.5)
≥ 12 months	218 (83.5)	216 (82.8)
p value	0.7366	
**Lifetime steroid use, *n* (%)**		
< 1 month or corticosteroid-naive	27 (10.3)	26 (10.0)
≥ 1 to < 12 months	12 (4.6)	14 (5.4)
≥ 12 months	222 (85.1)	221 (84.7)
p value	0.9162	
**Weight, kg**		
n	220	260
Mean (SD)	30.2 (13.5)	33.7 (18.7)
95% CI	28.4, 32.0	31.4, 36.0
Median	25.6	27.6
Min, max	11.8, 78.0	13.4, 106.1
p value	0.0179	
**Height, cm**		
n	187	259
Mean (SD)	121.9 (16.4)	130.8 (21.2)
95% CI	119.5, 124.3	128.2, 133.4
Median	121.0	126.8
Min, max	84.0, 177.0	90.1, 180.7
p value	< 0.0001	
**BMI, kg/m^2**		
n	186	183
Mean (SD)	19.0 (4.5)	18.1 (3.8)
95% CI	18.4, 19.7	17.6, 18.7
Median	17.6	17.1
Min, max	13.0, 40.1	10.8, 32.8
p value	0.0404	

CI confidence interval*, CINRG DNHS* Cooperative International Neuromuscular Research Group Duchenne Natural History Study, *SD* standard deviation, *STRIDE* Strategic Targeting of Registries and International Database of Excellence

Supplementary Table 6 Concomitant medication use (> 10% in medication class) in patients aged 5 years or older in the STRIDE Registry evaluable population

	**All patients** **N = 268**
**Patients receiving concomitant medication, *n* (%)**	261 (97.4)
**Corticosteroids**	244 (91.0)
Deflazacort	143 (53.4)
Dexamethasone	1 (0.4)
Fluticasone propionate	1 (0.4)
Mometasone furoate	1 (0.4)
Prednisolone	71 (26.5)
Prednisone	58 (21.6)
**Vitamin D and vitamin D analog**	217 (81.0)
Calcifediol	20 (7.5)
Calcitriol	2 (0.7)
Cholecalciferol	106 (39.6)
Ergocalciferol	5 (1.9)
Vitamin D (not otherwise specified)	9 (34.0)
**ACE inhibitors**	134 (50.0)
Captopril	2 (0.7)
Enalapril maleate	42 (15.7)
Lisinopril	10 (3.7)
Perindopril	42 (15.7)
Perindopril arginine	30 (11.2)
Perindopril erbumine	4 (1.5)
Ramipril	11 (4.1)
**Calcium**	68 (25.4)
Calcium	32 (11.9)
Calcium carbonate	33 (12.3)
Calcium carbonate; calcium lactate gluconate	1 (0.4)
Calcium citrate	1 (0.4)
Calcium phosphate	1 (0.4)
**Proton-pump inhibitors**	59 (22.0)
Esomeprazole	2 (0.7)
Esomeprazole magnesium	6 (2.2)
Lansoprazole	12 (4.5)
Omeprazole	35 (13.1)
Omeprazole magnesium	1 (0.4)
Omeprazole sodium	1 (0.4)
Pantoprazole	1 (0.4)
Pantoprazole sodium sesquihydrate	3 (1.1)
Rabeprazole sodium	1 (0.4)
**Beta-blocking agents, selective**	30 (11.2)
Bisoprolol	21 (7.8)
Bisoprolol fumarate	5 (1.9)
Bisoprolol succinate	3 (1.1)
Bisoprolol tartrate	1 (0.4)
**Osmotically acting laxatives**	27 (10.1)
Lactulose	1 (0.4)
Macrogol	1 (0.4)
Macrogol 3350	1 (0.4)

Macrogol 3350; potassium chloride; sodium bicarbonate	13 (4.9)
Macrogol 4000	9 (3.4)
Macrogol; potassium chloride; sodium bicarbonate; sodium chloride	1 (0.4)
Mannitol	1 (0.4)

Concomitant medications were coded using the WHO Drug Dictionary (September 2017). Concomitant medications are defined as any medications that patients started on or after the study treatment start date. Patients may have more than one medication per ATC system level 3 category and preferred term. At each level of patient summarization, a patient is counted once if the patient reported one or more medications. *ACE* angiotensin-converting enzyme, *ATC* Anatomical Therapeutic Classification, *STRIDE* Strategic Targeting of Registries and International Database of Excellence, *WHO* World Health Organization

Supplementary Table 7 TEAEs[a] experienced by patients aged ≥ 2 to < 5 in the as-treated population of the STRIDE Registry

	Corticosteroid use		
	**Yes** *N* **= 14**	**No** *N* **= 8**	**All** *N* **= 22**
**Number of TEAEs[a]**	13	1	14
**Patients with at least one of the following, *n* (%)**			
TEAE	6 (42.9)	1 (12.5)	7 (31.8)
TEAE related to ataluren	1 (7.1)	0 (0.0)	1 (4.5)
TEAE leading to discontinuation of ataluren	0 (0.0)	0 (0.0)	0 (0.0)
SAE	2 (14.3)	0 (0.0)	2 (9.1)
**TEAE with maximum severity[b]**			
Not reported	0 (0.0)	0 (0.0)	0 (0.0)
Unknown	0 (0.0)	0 (0.0)	0 (0.0)
Mild	4 (28.6)	1 (12.5)	5 (22.7)
Moderate	1 (7.1)	0 (0.0)	1 (4.5)
Severe	1 (7.1)	0 (0.0)	1 (4.5)
Life threatening	0 (0.0)	0 (0.0)	0 (0.0)
**Patients with at least one of the following, *n* (%)[c,d]**			
**Injury, poisoning and procedural complications**	**2 (14.3)**	**0 (0.0)**	**2 (9.1)**
**Infections and infestations**	**3 (21.4)**	**1 (12.5)**	**4 (18.2)**
Upper respiratory tract infection	2 (14.3)	0 (0.0)	2 (9.1)
**Investigations**	**2 (14.3)**	**0 (0.0)**	**2 (9.1)**

AE adverse event, *TEAE* treatment-emergent adverse event, *SAE* serious adverse event, *STRIDE* Strategic Targeting of Registries and International Database of Excellence

[a]TEAE is defined as any AE with a start date on or after the first date of ataluren use or worsened after initiation of ataluren treatment and does not necessarily have a causal relationship with ataluren. Events with missing severity are not reported

[b]For patients with two or more TEAEs, the event with the maximum severity was reported. The order of severity is 'not reported', 'unknown', 'mild', 'moderate', 'severe' and 'life threatening'

[c]AEs were coded using the Medical Dictionary for Regulatory Activities (version 20.1)

[d]A patient who reported at least one occurrence with the same preferred term was counted only once for that term.

Supplementary Table 8 Lipid profile of patients aged 5 years or older in the as-treated population of the STRIDE Registry (*N* = 284) at the first available and extreme assessments

		First available assessment result, *n* (%)			
	**Extreme assessment result, *n* (%)**[a]	**Normal**	**Abnormal, not clinically significant**[b]	**Abnormal, clinically significant**[b]	**Total**
**HDL**	Normal	94 (52.5)	12 (6.7)	0 (0.0)	106 (59.2)
	Abnormal, not clinically significant[b]	40 (22.3)	27 (15.1)	1 (0.6)	68 (38.0)
	Abnormal, clinically significant[b]	3 (1.7)	1 (0.6)	1 (0.6)	5 (2.8)
	Total	137 (76.5)	40 (22.3)	2 (1.1)	179 (100.0)
**LDL**	Normal	91 (54.2)	7 (4.2)	0 (0.0)	98 (58.3)
	Abnormal, not clinically significant[b]	35 (20.8)	26 (15.5)	2 (1.2)	63 (37.5)
	Abnormal, clinically significant[b]	2 (1.2)	2 (1.2)	3 (1.8)	7 (4.2)
	Total	128 (76.2)	35 (20.8)	5 (3.0)	168 (100.0)
**Triglycerides**	Normal	89 (40.8)	11 (5.0)	1 (0.5)	101 (46.3)
	Abnormal, not clinically significant[b]	54 (24.8)	48 (22.0)	1 (0.5)	103 (47.2)
	Abnormal, clinically significant[b]	7 (3.2)	2 (0.9)	5 (2.3)	14 (6.4)
	Total	150 (68.8)	61 (28.0)	7 (3.2)	218 (100.0)
**Total cholesterol**	Normal	98 (44.5)	10 (4.5)	1 (0.5)	109 (49.5)
	Abnormal, not clinically significant[b]	61 (27.7)	33 (15.0)	1 (0.5)	95 (43.2)
	Abnormal, clinically significant[b]	8 (3.6)	5 (2.3)	3 (1.4)	16 (7.3)
	Total	167 (75.9)	48 (21.8)	5 (2.3)	220 (100.0)

HDL high-density lipoprotein, *LDL* low-density lipoprotein, *STRIDE* Strategic Targeting of Registries and International Database of Excellence

[a]The extreme assessment result is the number and percentage of patients whose lipid profile shifted from the first available assessment to the most extreme assessment (worst value) during the period of ataluren use after the first available assessment

[b]The clinical significance of abnormal biochemistry results was determined by the patient's clinician

Supplementary Table 9 Lipid profile of patients aged 5 years or older in the as-treated population of the STRIDE Registry at the first available and extreme assessments who did not use corticosteroids (*N* = 25)

		First available assessment result, *n* (%)			
	**Extreme assessment result, *n* (%)**[a]	**Normal**	**Abnormal, not clinically significant**[b]	**Abnormal, clinically significant**[b]	**Total**
**HDL**	Normal	3 (21.4)	0 (0.0)	0 (0.0)	3 (21.4)
	Abnormal, not clinically significant[b]	5 (35.7)	4 (28.6)	1 (7.1)	10 (71.4)
	Abnormal, clinically significant[b]	0 (0.0)	1 (7.1)	0 (0.0)	1 (7.1)
	Total	8 (57.1)	5 (35.7)	1 (7.1)	14 (100.0)
**LDL**	Normal	7 (53.8)	1 (7.7)	0 (0.0)	8 (61.5)
	Abnormal, not clinically significant[b]	4 (30.8)	1 (7.7)	0 (0.0)	5 (38.5)
	Abnormal, clinically significant[b]	0 (0.0)	0 (0.0)	0 (0.0)	0 (0.0)
	Total	11 (84.6)	2 (15.4)	0 (0.0)	13 (100.0)
**Triglycerides**	Normal	8 (53.3)	0 (0.0)	0 (0.0)	8 (53.3)
	Abnormal, not clinically significant[b]	5 (33.3)	0 (0.0)	0 (0.0)	5 (33.3)
	Abnormal, clinically significant[b]	0 (0.0)	2 (13.3)	0 (0.0)	2 (13.3)
	Total	13 (86.7)	2 (13.3)	0 (0.0)	15 (100.0)
**Total cholesterol**	Normal	7 (46.7)	1 (6.7)	0 (0.0)	8 (53.3)
	Abnormal, not clinically significant[b]	4 (26.7)	1 (6.7)	0 (0.0)	5 (33.3)
	Abnormal, clinically significant[b]	1 (6.7)	1 (6.7)	0 (0.0)	2 (13.3)
	Total	12 (80.0)	3 (20.0)	0 (0.0)	15 (100.0)

HDL high-density lipoprotein, *LDL* low-density lipoprotein, *STRIDE* Strategic Targeting of Registries and International Database of Excellence

[a]The extreme assessment result is the number and percentage of patients shifted from the first available assessment to the most extreme assessment (worst value) during the period of ataluren use after the first available assessment

[b]The clinical significance of abnormal biochemistry results was determined by the clinician

Supplementary Table 10 ECG and heart rhythm results of patients aged 5 years or older in the as-treated population of the STRIDE Registry (*N* = 284) at the first available and extreme assessments

		First available assessment result, *n* (%)			
**ECG**	**Extreme assessment result, *n* (%)[a]**	**Normal**	**Abnormal, not clinically significant[b]**	**Abnormal, clinically significant[b]**	**Total**
	**Patients who used corticosteroids (*N* = 259)**				
	Normal	84 (48.0)	7 (4.0)	0 (0.0)	91 (52.0)
	Abnormal, not clinically significant[b]	47 (26.9)	32 (18.3)	1 (0.6)	80 (45.7)
	Abnormal, clinically significant[b]	3 (1.7)	1 (0.6)	0 (0.0)	4 (2.3)
	Total	134 (76.6)	40 (22.9)	1 (0.6)	175 (100.0)
	**Patients who did not use corticosteroids (*N* = 25)**				
	Normal	11 (73.3)	0 (0.0)	0 (0.0)	11 (73.3)
	Abnormal, not clinically significant[b]	4 (26.7)	0 (0.0)	0 (0.0)	4 (26.7)
	Abnormal, clinically significant[b]	0 (0.0)	0 (0.0)	0 (0.0)	0 (0.0)
	Total	15 (100)	0 (0.0)	0 (0.0)	15 (100.0)
**Heart rhythm**	**Extreme assessment result, *n* (%)[a]**	**Normal**	**Abnormal**	**Not calculable**	**Total**
	**Patients who used corticosteroids (*N* = 259)**				
	Normal	131 (78.0)	7 (4.2)	0 (0.0)	138 (82.1)
	Abnormal	23 (13.7)	7 (4.2)	0 (0.0)	30 (17.9)
	Total	154 (91.7)	14 (8.3)	0 (0.0)	168 (100.0)
	**Patients who did not use corticosteroids (*N* = 25)**				
	Normal	14 (87.5)	0 (0.0)	0 (0.0)	14 (87.5)
	Abnormal	2 (12.5)	0 (0.0)	0 (0.0)	2 (12.5)
	Total	16 (100.0)	0 (0.0)	0 (0.0)	16 (100.0)

ECG electrocardiogram, *STRIDE* Strategic Targeting of Registries and International Database of Excellence

[a]The extreme assessment result is the number and percentage of patients shifted from the first available assessment to the most extreme assessment (worst value) during the period of ataluren use after the first available assessment

[b]The clinical significance of abnormal ECG results was determined by the clinician

Supplementary Table 11 Hypertensive status and resting pulse rate of patients aged 5 years or older in the as-treated population of the STRIDE Registry (*N* = 284) at the first available and extreme assessments

		First available assessment result, *n* (%)			
**Hypertensive status**	**Extreme assessment result, *n* (%)[a]**	**Normal**	**Pre-hypertensive**	**Hypertensive**	**Total**
**Patients who used corticosteroids (*N* = 259)**					
	Normal	53 (22.6)	7 (3.0)	3 (1.3)	63 (26.8)
	Pre-hypertensive	21 (8.9)	5 (2.1)	7 (3.0)	33 (14.0)
	Hypertensive	65 (27.7)	24 (10.2)	50 (21.3)	139 (59.1)
	Total	139 (59.1)	36 (15.3)	60 (25.5)	235 (100.0)
**Patients who did not use corticosteroids (*N* = 25)**					
	Normal	5 (29.4)	0 (0.0)	0 (0.0)	5 (29.4)
	Pre-hypertensive	2 (11.8)	1 (5.9)	0 (0.0)	3 (17.6)
	Hypertensive	6 (35.5)	2 (11.8)	1 (5.9)	9 (52.9)
	Total	13 (76.5)	3 (17.6)	1 (5.9)	17 (100.0)
**Resting pulse rate, bpm**	**Extreme assessment result, *n* (%)[a]**	**Low**	**Normal**	**Elevated**	**Total**
**Patients who used corticosteroids (*N* = 259)**					
Shift to the highest					
	Low	0 (0.0)	0 (0.0)	0 (0.0)	0 (0.0)
	Normal	4 (2.4)	140 (84.8)	4 (2.4)	148 (89.7)
	Elevated	0 (0.0)	13 (7.9)	4 (2.4)	17 (10.3)
	Total	4 (2.4)	153 (92.7)	8 (4.8)	165 (100.0)
Shift to the lowest					
	Low	1 (0.6)	18 (10.9)	0 (0.0)	19 (11.5)
	Normal	3 (1.8)	133 (80.6)	6 (3.6)	142 (86.1)
	Elevated	0 (0.0)	2 (1.2)	2 (1.2)	4 (2.4)
	Total	4 (2.4)	153 (92.7)	8 (4.8)	165 (100.0)
**Patients who did not use corticosteroids (*N* = 25)**					
Shift to the highest					
	Low	0 (0.0)	0 (0.0)	0 (0.0)	0 (0.0)
	Normal	2 (11.8)	13 (76.5)	1 (5.9)	16 (94.1)

	Elevated	0 (0.0)	1 (5.9)	0 (0.0)	1 (5.9)
	Total	2 (11.8)	14 (82.4)	1 (5.9)	17 (100.0)
Shift to the lowest					
	Low	1 (5.9)	2 (11.8)	1 (5.9)	4 (23.5)
	Normal	1 (5.9)	12 (70.6)	0 (0.0)	13 (76.5)
	Elevated	0 (0.0)	0 (0.0)	0 (0.0)	0 (0.0)
	Total	2 (11.8)	14 (82.4)	1 (5.9)	17 (100.0)

STRIDE Strategic Targeting of Registries and International Database of Excellence

[a]The extreme assessment result is the number and percentage of patients shifted from the first available assessment to the most extreme assessment (worst value) during the period of ataluren use after the first available assessment

Supplementary Table 12 Hepatic enzyme and renal profile of patients aged 5 years or older in the as-treated population of the STRIDE Registry (N = 284) at the first available and extreme assessments

		**First available assessment result, *n* (%)**			
	**Extreme assessment result, *n* (%)**[a]	**Normal**	**Abnormal, not clinically significant**[b]	**Abnormal, clinically significant**[b]	**Total**
**AST**	Normal	1 (0.5)	1 (0.5)	0 (0.0)	2 (1.1)
	Abnormal, not clinically significant[b]	6 (3.3)	123 (67.2)	17 (9.3)	146 (79.8)
	Abnormal, clinically significant[b]	0 (0.0)	20 (10.9)	15 (8.2)	35 (19.1)
	Total	7 (3.8)	144 (78.7)	32 (17.5)	183 (100.0)
**ALT**	Normal	1 (0.4)	3 (1.3)	0 (0.0)	4 (1.8)
	Abnormal, not clinically significant[b]	2 (0.9)	161 (71.9)	17 (7.6)	180 (80.4)
	Abnormal, clinically significant[b]	0 (0.0)	24 (10.7)	16 (7.1)	40 (17.9)
	Total	3 (1.3)	188 (83.9)	33 (14.7)	224 (100.0)
**GGT**	Normal	103 (53.9)	8 (4.2)	0 (0.0)	111 (58.1)
	Abnormal, not clinically significant[b]	58 (30.4)	19 (9.9)	0 (0.0)	77 (40.3)
	Abnormal, clinically significant[b]	1 (0.5)	2 (1.0)	0 (0.0)	3 (1.6)
	Total	162 (84.8)	29 (15.2)	0 (0.0)	191 (100.0)
**Total bilirubin**	Normal	129 (77.2)	7 (4.2)	0 (0.0)	136 (81.4)
	Abnormal, not clinically significant[b]	20 (12.0)	9 (5.4)	1 (0.6)	30 (18.0)
	Abnormal, clinically significant[b]	1 (0.6)	0 (0.0)	0 (0.0)	1 (0.6)
	Total	150 (89.8)	16 (9.6)	1 (0.6)	167 (100.0)
**Serum creatinine**	Normal	23 (9.9)	7 (3.0)	1 (0.4)	31 (13.3)
	Abnormal, not clinically significant[b]	55 (23.6)	135 (57.9)	2 (0.9)	192 (82.4)
	Abnormal, clinically significant[b]	3 (1.3)	7 (3.0)	0 (0.0)	10 (4.3)
	Total	81 (34.8)	149 (63.9)	3 (1.3)	233 (100.0)
**BUN**	Normal	117 (68.4)	3 (1.8)	0 (0.0)	120 (70.2)
	Abnormal, not clinically significant[b]	41 (24.0)	10 (5.8)	0 (0.0)	51 (29.8)
	Abnormal, clinically significant[b]	0 (0.0)	0 (0.0)	0 (0.0)	0 (0.0)
	Total	158 (92.4)	13 (7.6)	0 (0.0)	171 (100.0)

**Cystatin C**	Normal	63 (75.9)	2 (2.4)	0 (0.0)	65 (78.3)
	Abnormal, not clinically significant[b]	11 (13.3)	6 (7.2)	0 (0.0)	17 (20.5)
	Abnormal, clinically significant[b]	1 (1.2)	0 (0.0)	0 (0.0)	1 (1.2)
	Total	75 (90.4)	8 (9.6)	0 (0.0)	83 (100.0)

ALT alanine transaminase, *AST* aspartate aminotransferase, *BUN* blood urea nitrogen, *GGT* gamma-glutamyl transferase, *STRIDE* Strategic Targeting of Registries and International Database of Excellence

[a]The extreme assessment result is the number and percentage of patients shifted from the first available assessment to the most extreme assessment (worst value) during the period of ataluren use after the first available assessment

[b]The clinical significance of abnormal biochemistry results was determined by the clinician

Supplementary Table 13 Lipid profile of patients aged ≥ 2 to < 5 years in the as-treated population of the STRIDE Registry (*N* = 22) at the first available and extreme assessments

		First available assessment result, *n* (%)			
	**Extreme assessment result, *n* (%)**[a]	**Normal**	**Abnormal, not clinically significant**[b]	**Abnormal, clinically significant**[b]	**Total**
**HDL**	Normal	5 (45.5)	0 (0.0)	0 (0.0)	5 (45.5)
	Abnormal, not clinically significant[b]	2 (18.2)	2 (18.2)	0 (0.0)	4 (36.4)
	Abnormal, clinically significant[b]	1 (9.1)	1 (9.1)	0 (0.0)	2 (18.2)
	Total	8 (88.9)	3 (27.3)	0 (0.0)	11 (100.0)
**LDL**	Normal	5 (55.6)	0 (0.0)	0 (0.0)	5 (55.6)
	Abnormal, not clinically significant[b]	3 (33.3)	1 (11.1)	0 (0.0)	4 (44.4)
	Abnormal, clinically significant[b]	0 (0.0)	0 (0.0)	0 (0.0)	0 (0.0)
	Total	8 (88.9)	1 (11.1)	0 (0.0)	9 (100.0)
**Triglycerides**	Normal	6 (54.5)	0 (0.0)	0 (0.0)	6 (54.5)
	Abnormal, not clinically significant[b]	4 (36.4)	0 (0.0)	0 (0.0)	4 (36.4)
	Abnormal, clinically significant[b]	1 (9.1)	0 (0.0)	0 (0.0)	1 (9.1)
	Total	11 (100.0)	0 (0.0)	0 (0.0)	11 (100.0)
**Total cholesterol**	Normal	7 (58.3)	0 (0.0)	0 (0.0)	7 (58.3)
	Abnormal, not clinically significant[b]	4 (33.3)	1 (8.3)	0 (0.0)	5 (41.7)
	Abnormal, clinically significant[b]	0 (0.0)	0 (0.0)	0 (0.0)	0 (0.0)
	Total	11 (91.7)	1 (8.3)	0 (0.0)	12 (100.0)

HDL high-density lipoprotein, *LDL* low-density lipoprotein, *STRIDE* Strategic Targeting of Registries and International Database of Excellence

[a]The extreme assessment result is the number and percentage of patients shifted from the first available assessment to the most extreme assessment (worst value) during the period of ataluren use after the first available assessment

[b]The clinical significance of abnormal biochemistry results was determined by the clinician

Supplementary Table 14 Lipid profile of patients aged ≥ 2 to < 5 years in the as-treated population of the STRIDE Registry at the first available and extreme assessments who were not receiving corticosteroids (*N* = 8)

		First available assessment result, *n* (%)			
	**Extreme assessment result, *n* (%)**[a]	**Normal**	**Abnormal, not clinically significant**[b]	**Abnormal, clinically significant**[b]	**Total**
**HDL**	Normal	1 (33.3)	0 (0.0)	0 (0.0)	1 (33.3)
	Abnormal, not clinically significant[b]	1 (33.3)	1 (33.3)	0 (0.0)	2 (66.7)
	Abnormal, clinically significant[b]	0 (0.0)	0 (0.0)	0 (0.0)	0 (0.0)
	Total	2 (66.7)	1 (33.3)	0 (0.0)	3 (100.0)
**LDL**	Normal	1 (33.3)	0 (0.0)	0 (0.0)	1 (33.3)
	Abnormal, not clinically significant[b]	1 (33.3)	1 (33.3)	0 (0.0)	2 (66.7)
	Abnormal, clinically significant[b]	0 (0.0)	0 (0.0)	0 (0.0)	0 (0.0)
	Total	2 (66.7)	1 (33.3)	0 (0.0)	3 (100.0)
**Triglycerides**	Normal	0 (0.0)	0 (0.0)	0 (0.0)	0 (0.0)
	Abnormal, not clinically significant[b]	3 (100.0)	0 (0.0)	0 (0.0)	3 (100.0)
	Abnormal, clinically significant[b]	0 (0.0)	0 (0.0)	0 (0.0)	0 (0.0)
	Total	3 (100.0)	0 (0.0)	0 (0.0)	3 (100.0)
**Total cholesterol**	Normal	2 (50.0)	0 (0.0)	0 (0.0)	2 (50.0)
	Abnormal, not clinically significant[b]	1 (25.0)	1 (25.0)	0 (0.0)	2 (50.0)
	Abnormal, clinically significant[b]	0 (0.0)	0 (0.0)	0 (0.0)	0 (0.0)
	Total	3 (75.0)	1 (25.0)	0 (0.0)	4 (100.0)

HDL high-density lipoprotein, *LDL* low-density lipoprotein, *STRIDE* Strategic Targeting of Registries and International Database of Excellence

[a]The extreme assessment result is the number and percentage of patients shifted from the first available assessment to the most extreme assessment (worst value) during the period of ataluren use after the first available assessment

[b]The clinical significance of abnormal biochemistry results was determined by the clinician

Supplementary Table 15 ECG and heart rhythm results of patients aged ≥ 2 to < 5 years in the as-treated population of the STRIDE Registry (*N* = 22) at the first available and extreme assessments

		First available assessment result, *n* (%)			
**ECG**	**Extreme assessment result, *n* (%)[a]**	**Normal**	**Abnormal, not clinically significant[b]**	**Abnormal, clinically significant[b]**	**Total**
	**Patients who used corticosteroids (*N* = 14)**				
	Normal	8 (80.0)	2 (20.0)	0 (0.0)	10 (100.0)
	Abnormal, not clinically significant[b]	0 (0.0)	0 (0.0)	0 (0.0)	0 (0.0)
	Abnormal, clinically significant[b]	0 (0.0)	0 (0.0)	0 (0.0)	0 (0.0)
	Total	8 (80.0)	2 (20.0)	0 (0.0)	10 (100.0)
	**Patientswho did not use corticosteroids (*N* = 8)**				
	Normal	3 (100.0)	0 (0.0)	0 (0.0)	3 (100.0)
	Abnormal, not clinically significant[b]	0 (0.0)	0 (0.0)	0 (0.0)	0 (0.0)
	Abnormal, clinically significant[b]	0 (0.0)	0 (0.0)	0 (0.0)	0 (0.0)
	Total	3 (100.0)	0 (0.0)	0 (0.0)	3 (100.0)
**Heart rhythm**	**Extreme assessment result, *n* (%)[a]**	**Normal**	**Abnormal**	**Not calculable**	**Total**
	**Patients who used corticosteroids (*N* = 14)**				
	Normal	10 (100.0)	0 (0.0)	0 (0.0)	10 (100.0)
	Abnormal	0 (0.0)	0 (0.0)	0 (0.0)	0 (0.0)
	Total	10 (100.0)	0 (0.0)	0 (0.0)	10 (100.0)
	**Patients who did not use corticosteroids (*N* = 8)**				
	Normal	2 (66.7)	1 (33.3)	0 (0.0)	3 (100.0)
	Abnormal	0 (0.0)	0 (0.0)	0 (0.0)	0 (0.0)
	Total	2 (66.7)	1 (33.3)	0 (0.0)	3 (100.0)

ECG electrocardiogram, *STRIDE* Strategic Targeting of Registries and International Database of Excellence

[a]The extreme assessment result is the number and percentage of patients shifted from the first available assessment to the most extreme assessment (worst value) during the period of ataluren use after the first available assessment

[b]The clinical significance of abnormal ECG results was determined by the clinician

Supplementary Table 16 Hypertensive status and resting pulse rate of patients aged ≥ 2 to < 5 years in the as-treated population of the STRIDE Registry (*N* = 22) at the first available and extreme assessments

		First available assessment result, *n* (%)			
**Hypertensive status**	**Extreme assessment result, *n* (%)[a]**	**Normal**	**Pre-hypertensive**	**Hypertensive**	**Total**
**Patients who used corticosteroids (*N* = 14)**					
	Normal	1 (10.0)	1 (10.0)	0 (0.0)	2 (20.0)
	Pre-hypertensive	2 (20.0)	1 (10.0)	0 (0.0)	3 (30.0)
	Hypertensive	2 (20.0)	1 (10.0)	2 (20.0)	5 (50.0)
	Total	5 (50.0)	3 (30.0)	2 (20.0)	10 (100.0)
**Patients who did not use corticosteroids (*N* = 8)**					
	Normal	1 (20.0)	2 (40.0)	0 (0.0)	3 (60.0)
	Pre-hypertensive	0 (0.0)	0 (0.0)	0 (0.0)	0 (0.0)
	Hypertensive	2 (40.0)	0 (0.0)	0 (0.0)	2 (40.0)
	Total	3 (60.0)	2 (40.0)	0 (0.0)	5 (100.0)
**Resting pulse rate, bpm**	**Extreme assessment result, *n* (%)[a]**	**Low**	**Normal**	**Elevated**	**Total**
**Patients who used corticosteroids (*N* = 14)**					
Shift to the highest					
	Low	0 (0.0)	0 (0.0)	0 (0.0)	0 (0.0)
	Normal	0 (0.0)	7 (100.0)	0 (0.0)	7 (100.0)
	Elevated	0 (0.0)	0 (0.0)	0 (0.0)	0 (0.0)
	Total	0 (0.0)	7 (100.0)	0 (0.0)	7 (100.0)
Shift to the lowest					
	Low	0 (0.0)	0 (0.0)	0 (0.0)	0 (0.0)
	Normal	0 (0.0)	7 (100.0)	0 (0.0)	7 (100.0)
	Elevated	0 (0.0)	0 (0.0)	0 (0.0)	0 (0.0)
	Total	0 (0.0)	7 (100.0)	0 (0.0)	7 (100.0)
**Patients who did not use corticosteroids (*N* = 8)**					
Shift to the highest					
	Low	0 (0.0)	0 (0.0)	0 (0.0)	0 (0.0)
	Normal	0 (0.0)	3 (100.0)	0 (0.0)	3 (100.0)

	Elevated	0 (0.0)	0 (0.0)	0 (0.0)	0 (0.0)
	Total	0 (0.0)	3 (100.0)	0 (0.0)	3 (100.0)
Shift to the lowest					
	Low	0 (0.0)	0 (0.0)	0 (0.0)	0 (0.0)
	Normal	0 (0.0)	3 (100.0)	0 (0.0)	3 (100.0)
	Elevated	0 (0.0)	0 (0.0)	0 (0.0)	0 (0.0)
	Total	0 (0.0)	3 (100.0)	0 (0.0)	3 (100.0)

STRIDE Strategic Targeting of Registries and International Database of Excellence

[a]The extreme assessment result is the number and percentage of patients shifted from the first available assessment to the most extreme assessment (worst value) during the period of ataluren use after the first available assessment

Supplementary Table 17 Hepatic enzyme and renal profile of patients aged ≥ 2 to < 5 years in the as-treated population of the STRIDE Registry (*N* = 22) at the first available and extreme assessments

		First available assessment result, *n* (%)			
	**Extreme assessment result, *n* (%)**[a]	**Normal**	**Abnormal, not clinically significant**[b]	**Abnormal, clinically significant**[b]	**Total**
**AST**	Normal	0 (0.0)	0 (0.0)	0 (0.0)	0 (0.0)
	Abnormal, not clinically significant[b]	0 (0.0)	9 (64.3)	2 (14.3)	11 (78.6)
	Abnormal, clinically significant[b]	0 (0.0)	1 (7.1)	2 (14.3)	3 (21.4)
	Total	0 (0.0)	10 (71.4)	4 (28.6)	14 (100.0)
**ALT**	Normal	0 (0.0)	0 (0.0)	0 (0.0)	0 (0.0)
	Abnormal, not clinically significant[b]	0 (0.0)	11 (68.8)	2 (12.5)	13 (81.3)
	Abnormal, clinically significant[b]	0 (0.0)	1 (6.3)	2 (12.5)	3 (18.8)
	Total	0 (0.0)	12 (75.0)	4 (25.0)	16 (100.0)
**GGT**	Normal	8 (72.7)	0 (0.0)	0 (0.0)	8 (72.7)
	Abnormal, not clinically significant[b]	1 (9.1)	2 (18.2)	0 (0.0)	3 (27.3)
	Abnormal, clinically significant[b]	0 (0.0)	0 (0.0)	0 (0.0)	0 (0.0)
	Total	9 (81.8)	2 (18.2)	0 (0.0)	11 (100.0)
**Total bilirubin**	Normal	8 (80.0)	0 (0.0)	0 (0.0)	8 (80.0)
	Abnormal, not clinically significant[b]	1 (10.0)	1 (10.0)	0 (0.0)	2 (20.0)
	Abnormal, clinically significant[b]	0 (0.0)	0 (0.0)	0 (0.0)	0 (0.0)
	Total	9 (90.0)	1 (10.0)	0 (0.0)	10 (100.0)
**Serum creatinine**	Normal	5 (29.4)	0 (0.0)	0 (0.0)	5 (29.4)
	Abnormal, not clinically significant[b]	3 (17.6)	9 (52.9)	0 (0.0)	12 (70.6)
	Abnormal, clinically significant[b]	0 (0.0)	0 (0.0)	0 (0.0)	0 (0.0)
	Total	8 (47.1)	9 (52.9)	0 (0.0)	17 (100.0)
**BUN**	Normal	13 (92.9)	0 (0.0)	0 (0.0)	13 (92.9)
	Abnormal, not clinically significant[b]	1 (7.1)	0 (0.0)	0 (0.0)	1 (7.1)
	Abnormal, clinically significant[b]	0 (0.0)	0 (0.0)	0 (0.0)	0 (0.0)
	Total	14 (100.0)	0 (0.0)	0 (0.0)	14 (100.0)

**Cystatin C**	Normal	2 (66.7)	0 (0.0)	0 (0.0)	2 (66.7)
	Abnormal, not clinically significant[b]	1 (33.3)	0 (0.0)	0 (0.0)	1 (33.3)
	Abnormal, clinically significant[b]	0 (0.0)	0 (0.0)	0 (0.0)	0 (0.0)
	Total	3 (100.0)	0 (0.0)	0 (0.0)	3 (100.0)

ALT alanine transaminase, *AST* aspartate aminotransferase, *BUN* blood urea nitrogen, *GGT* gamma-glutamyl transferase, *STRIDE* Strategic Targeting of Registries and International Database of Excellence

[a]The extreme assessment result is the number and percentage of patients shifted from the first available assessment to the most extreme assessment (worst value) during the period of ataluren use after the first available assessment

[b]The clinical significance of abnormal biochemistry results was determined by the clinician

Supplementary Table 18 Shift table of body weight characteristics (from first to extreme assessments) for the propensity-score matched STRIDE Registry and CINRG DNHS populations

	**First available assessment result, *N* (%)**									
	**Underweight**		**Normal weight**		**Overweight**		**Obese**		**Total**	
**Extreme assessment result, *n* (%)**[a]	**STRIDE (*n* = 211)**	**CINRG (*n* = 167)**	**STRIDE (*n* = 211)**	**CINRG (*n* = 167)**	**STRIDE (*n* = 211)**	**CINRG (*n* = 167)**	**STRIDE (*n* = 211)**	**CINRG (*n* = 167)**	**STRIDE (*n* = 211)**	**CINRG (*n* = 167)**
**Underweight**	4 (1.9)	2 (1.2)	2 (0.9)	2 (1.2)	0 (0.0)	1 (0.6)	0 (0.0)	0 (0.0)	6 (2.8)	5 (3.0)
**Normal weight**	5 (2.4)	5 (3.0)	81 (38.4)	53 (31.7)	5 (2.4)	0 (0.0)	0 (0.0)	0 (0.0)	91 (43.1)	58 (34.7)
**Overweight**	0 (0.0)	0 (0.0)	27 (12.8)	27 (16.2)	21 (10.0)	17 (10.2)	2 (0.9)	2 (1.2)	50 (23.7)	46 (27.5)
**Obese**	0 (0.0)	0 (0.0)	11 (5.2)	10 (6.0)	19 (9.0)	15 (9.0)	34 (16.1)	33 (19.8)	64 (30.3)	58 (34.7)
**Total**	9 (4.3)	7 (4.2)	121 (57.3)	92 (55.1)	45 (21.3)	33 (19.8)	36 (17.1)	35 (21.0)	211 (100)	167 (100)

Body weight is classified and determined based on BMI percentiles, which have been developed and issued by the US Centers for Disease Control and Prevention. The first available assessment is used as the base comparison. For non-ambulatory patients, the last qualified assessment should be no later than loss of ambulation. *BMI* body mass index, *CINRG DNHS* Cooperative International Neuromuscular Research Group Duchenne Natural History Study, *STRIDE* Strategic Targeting of Registries and International Database of Excellence

[a]The extreme result is the worst case during ataluren treatment after first available assessment

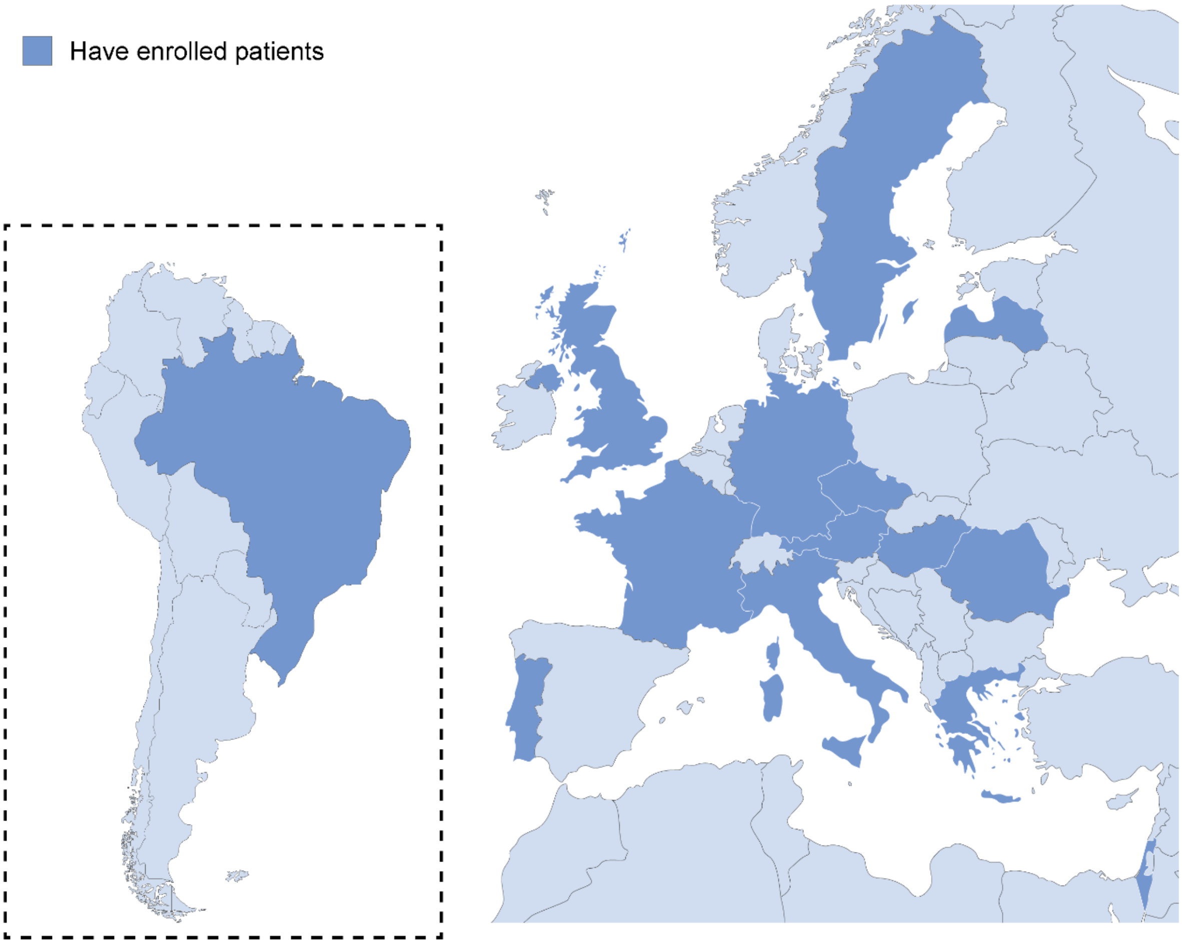

Supplementary Fig. 1 Countries with patients enrolled in the STRIDE Registry. All included patients provided informed consent. *STRIDE* Strategic Targeting of Registries and International Database of Excellence

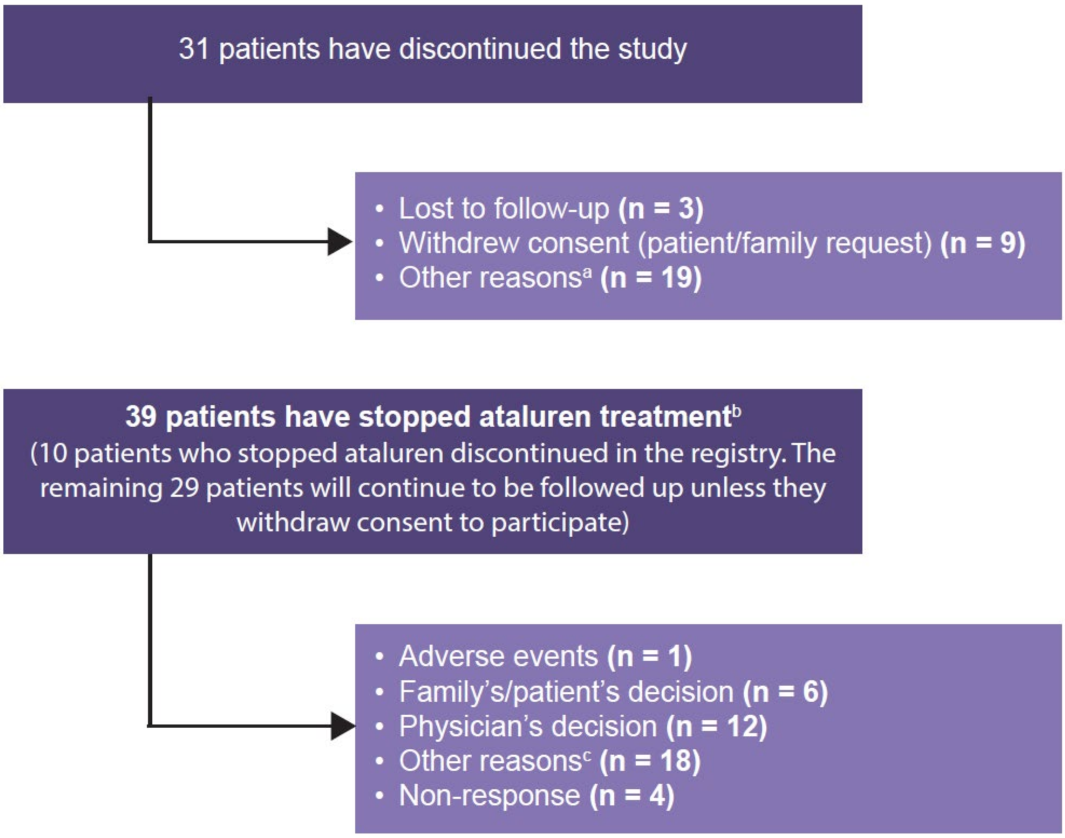

Supplementary Fig. 2 Study discontinuation and ataluren cessation for patients aged 5 years or older in the evaluable population (*N* = 268)

[a]Other reasons include: family decided to stop the treatment and participation in the study; loss of ambulation; the patient chose to stop ataluren

[b]Patients can have multiple reasons to stop or change dose, hence the number of reasons totals 41 not 39

[c]Other reasons include: loss of ambulation; therapeutic window due to nasal congestion requested by mother; ataluren no longer prescribable/reimbursable because of loss of independent ambulation; stop criterion; stopped by authorities

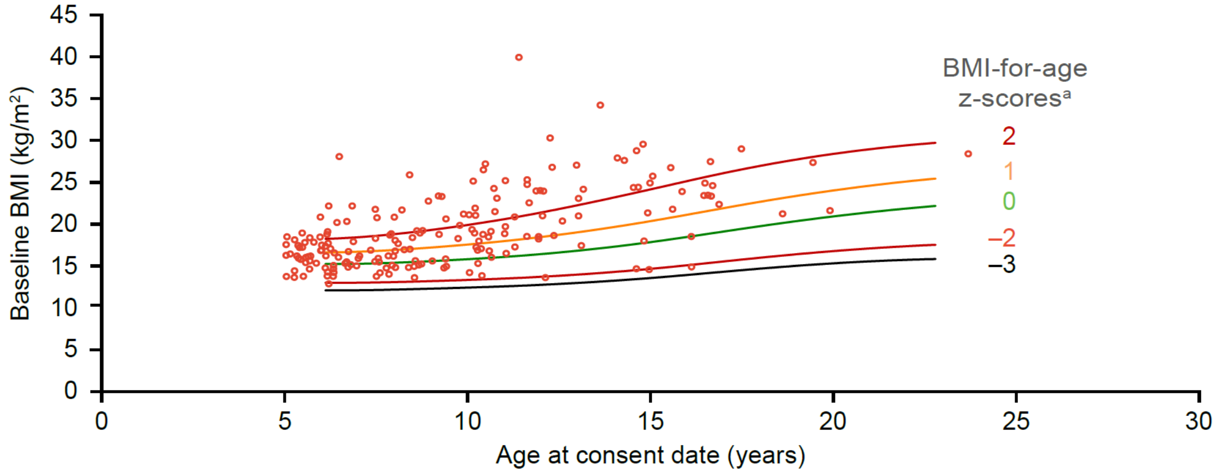

Supplementary Fig. 3 Distribution of BMI for STRIDE Registry patients in the evaluable population aged 5 years or older, by patient age at consent date. *BMI* body mass index, *STRIDE* Strategic Targeting of Registries and International Database of Excellence
[a]The lines indicate z-scores of BMI-for-age according to the growth reference (boys aged 5–19 years) provided by the World Health Organization [1]

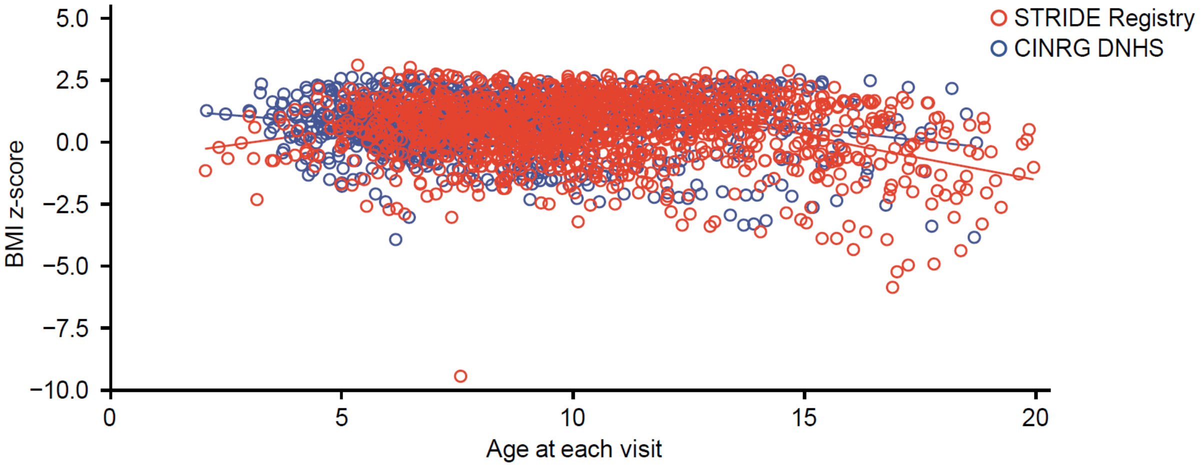

Supplementary Fig. 4 BMI z-score for patients in propensity-score matched STRIDE Registry and CINRG DNHS populations. Patients without a second assessment or the duration between the first and last assessments were < 40 weeks apart were excluded for the analysis. *BMI* body mass index, *CINRG DNHS* Cooperative International Neuromuscular Research Group Duchenne Natural History Study, *STRIDE* Strategic Targeting of Registries and International Database of Excellence

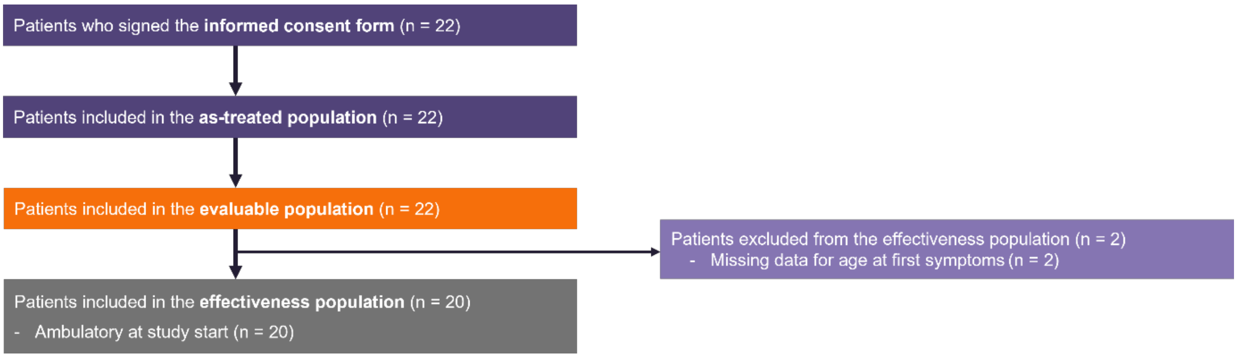

Supplementary Fig. 5 Patient disposition in the < 2 to > 5 year old STRIDE Registry analysis population. *DMD* Duchenne muscular dystrophy, *NBS* newborn screening, *PND* prenatal diagnosis, *STRIDE* Strategic Targeting of Registries and International Database of Excellence.

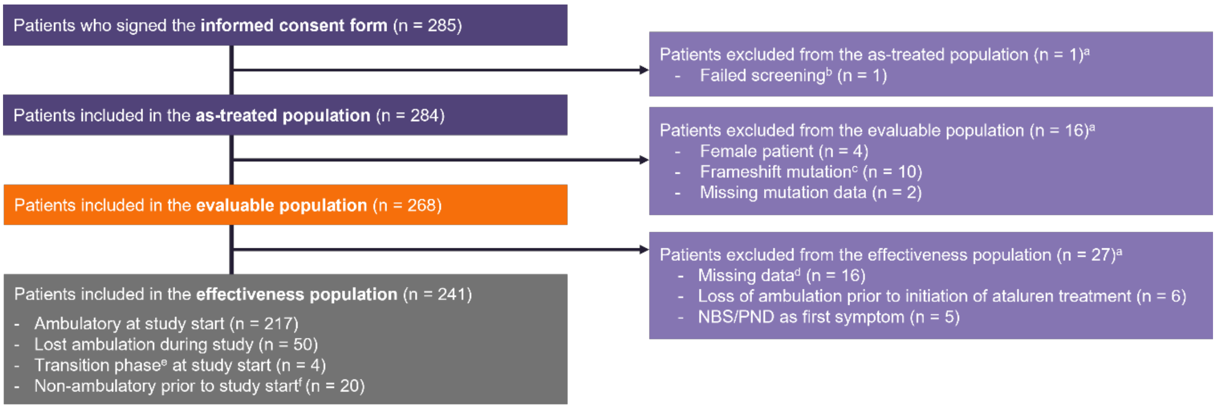

Supplementary Fig. 6 Patient disposition in the ≥ 5 year old STRIDE Registry analysis population. *DMD* Duchenne muscular dystrophy, *NBS* newborn screening, *PND* prenatal diagnosis, *STRIDE* Strategic Targeting of Registries and International Database of Excellence.

[a]Patients may have been grouped in more than one category

[b]Screening failure owing to a frameshift mutation

[c]Ataluren is not indicated in these patients; ataluren is indicated for the treatment of ambulatory patients with DMD resulting from a nonsense mutation in the dystrophin gene. Patients who do not have a nonsense mutation should not receive ataluren [2]

[e]Patients were in the transition phase if they completed the first 10-m walk/run test in ≥ 30 seconds

[f]Non-ambulatory patients were defined as such if using a wheelchair fulltime or bedridden; patients who were non-ambulatory “prior to study start” were all ambulatory at ataluren initiation in previous clinical trials

References

1. World Health Organization data, available from https://www.who.int/growthref/who2007_bmi_for_age/en/. Accessed May 23, 2022

2. European Medicines Agency. Translarna™ summary of product characteristics. Available from: www.ema.europa.eu/en/documents/product-information/translarna-epar-product-information_en.pdf. Accessed April 15, 2022
